# Bik promotes proteasomal degradation to control low-grade inflammation

**DOI:** 10.1172/JCI170594

**Published:** 2023-12-19

**Authors:** Yohannes A. Mebratu, Jane T. Jones, Congjian Liu, Zerihun H. Negasi, Mizanur Rahman, Joselyn Rojas-Quintero, George T. O’Connor, Wei Gao, Josée Dupuis, Michael H. Cho, Augusto A. Litonjua, Scott Randell, Yohannes Tesfaigzi

**Affiliations:** 1 Brigham and Women’s Hospital, Division of Pulmonary and Critical Medicine, Harvard Medical School, Boston, Massachusetts, USA.; 2 University Geisel School of Medicine, Department of Microbiology and Immunology, Dartmouth, Hanover, New Hampshire, USA.; 3 Pulmonary Center, Boston University School of Medicine, Boston, Massachusetts, USA.; 4 National Heart, Lung, and Blood Institute’s (NHLBI’s) Framingham Heart Study, Framingham, Massachusetts, USA.; 5 Department of Biostatistics, Boston University School of Public Health, Boston, Massachusetts, USA.; 6 Department of Epidemiology, Biostatistics, and Occupational Health, McGill University, Montreal, Canada.; 7 Channing Division of Network Medicine, Brigham and Women’s Hospital, Boston, Massachusetts, USA.; 8 Division of Pediatric Pulmonary Medicine, University of Rochester Medical Center, Rochester, New York, USA.; 9 Marsico Lung Institute, UNC School of Medicine, Chapel Hill, North Carolina, USA.

**Keywords:** Inflammation, Apoptosis, NF-kappaB, Peptides

## Abstract

Although chronic low-grade inflammation does not cause immediate clinical symptoms, over the longer term, it can enhance other insults or age-dependent damage to organ systems and thereby contribute to age-related disorders, such as respiratory disorders, heart disease, metabolic disorders, autoimmunity, and cancer. However, the molecular mechanisms governing low-level inflammation are largely unknown. We discovered that Bcl-2–interacting killer (Bik) deficiency causes low-level inflammation even at baseline and the development of spontaneous emphysema in female but not male mice. Similarly, a single nucleotide polymorphism that reduced Bik levels was associated with increased inflammation and enhanced decline in lung function in humans. Transgenic expression of Bik in the airways of *Bik*-deficient mice inhibited allergen- or LPS-induced lung inflammation and reversed emphysema in female mice. Bik deficiency increased nuclear but not cytosolic p65 levels because Bik, by modifying the BH4 domain of Bcl-2, interacted with regulatory particle non-ATPase 1 (RPN1) and RPN2 and enhanced proteasomal degradation of nuclear proteins. Bik deficiency increased inflammation primarily in females because Bcl-2 and Bik levels were reduced in lung tissues and airway cells of female compared with male mice. Therefore, controlling low-grade inflammation by modifying the unappreciated role of Bik and Bcl-2 in facilitating proteasomal degradation of nuclear proteins may be crucial in treating chronic age-related diseases.

## Abstract 2

<p>The cell death regulatory Bik binds to nuclear Bcl-2 to modify protein stability and inflammation.</p>

## Introduction

Inflammation is part of many important defense mechanisms that are necessary to protect the host from both infections and other environmental insults. Several highly conserved pathways orchestrate the onset and progression of inflammation to ensure an effective and adequately controlled response to safely remove pathogens and damaged tissue components and facilitate tissue repair (1). Once insults are cleared, negative feedback mechanisms such as antiinflammatory mediators, resolve inflammation. If the resolution process is dysfunctional, chronic inflammation eventually causes scarring and organ damage and failure, as observed in many prevalent diseases, including chronic obstructive pulmonary disease (COPD), atherosclerosis, and diabetes and their associated complications (2).

Inflammation induced by pathogens and other environmental injuries is often extensive and acute; however, production of ROS or of autoantibodies over longer periods can cause a sustained low-grade chronic inflammation (3). Low-grade inflammation does not generally cause immediate clinical symptoms, but over the longer term, it can serve as a primer or enhancer of other insults or of age-dependent damage to organ systems. Therefore, low-grade inflammation contributes to age-related disorders, such as heart disease, respiratory disorders, neurodegeneration, metabolic disorders, autoimmunity, and cancer (4–6). The aging process is associated with a 2- to 4-fold increase in circulating inflammatory mediators (4, 7). Despite the importance of chronic low-grade inflammation, the molecular and cellular mechanisms governing low-level inflammation and its effect on organisms have not been explored as extensively as has been inflammation induced by engagement of TNF receptors, TLRs, or pattern recognition receptors (PRRs) (8).

Inflammation in the airway epithelium is predominantly regulated by the proinflammatory transcription factor/signaling pathway NF-κB (9–12) and promotes asthma (13) or COPD (14) development. Phosphorylation of IκBα by IKKβ leads to degradation by the 26S proteasome so that p65 can translocate into the nucleus and initiate transcription of inflammatory genes (15). Posttranslational modifications, such as phosphorylation, acetylation, methylation, and ubiquitination, fine-tune transcriptional activity, potency, and selectivity of NF-κB to interact and initiate transcription from specific DNA-responsive elements (15).

The Bcl-2 family proteins have classically been thought to function as regulators of cell death, but members of this family also regulate inflammation. The BH3-only protein, Bid, interacts with NOD1, NOD2, and the IκB kinase (IKK) complex and enhances the signaling of the IKK complex, which regulates downstream inflammatory responses and bowel disease (16). We found that Noxa, another BH3-only protein, suppresses degradation of ubiquitinated IκBα and thereby inhibits allergic inflammation (17). Furthermore, Bcl-2 itself has been shown to dampen NF-κB transcriptional activity via its BH2 and BH4 domains (18), although the mechanisms are unknown. The Bcl-2–interacting killer (*BIK*) gene is the founding member of the BH3-only proapoptotic proteins that, when increased in expression, mainly localizes to the endoplasmic reticulum (19). In human airway epithelial cells (HAECs), IFN-γ requires Bik to facilitate resolution of inflammation-induced airway epithelial cell (AEC) hyperplasia by inducing cell death of proliferating epithelial cells (20). Hyperplastic and metaplastic mucous cells are sustained by cigarette smoke (CS) exposure because CS reduces Bik expression (21), and in humans, Bik levels remain reduced in former smokers with chronic bronchitis (21).

Because Bik has a high affinity to Bcl-2 (22–24) and Bcl-2 was shown to affect inflammation, we investigated whether Bik expressed at baseline may have a role in affecting low-level inflammation in airway cells. The present study first evaluated whether Bik deficiency is associated with low-grade inflammation and investigated the molecular mechanisms of how this interaction affects NF-κB. In humans, a genetic variant in the intronic region of the *BIK* gene introduces an interferon regulatory transcription factor-1–binding (IRF-1–binding) site that suppresses Bik levels by 2-fold. Due to its high-affinity binding to Bcl-2 in the nucleus, Bik facilitated the interaction with regulatory particle non-ATPase 1 (RPN1) and RPN2, 2 subunits of the 19S regulatory cap of the 20S proteasome, to promote degradation of many proteins, including nuclear, but not cytosolic, p65. Persistent low-level increase in p65 and inflammation in Bik-deficient cells resulted in emphysematous lungs. Restoring Bik expression in the airways prevented the development of emphysema. Collectively, our studies demonstrate a mechanism by which nuclear p65 levels are regulated to suppress low-level inflammation.

## Results

### Bik deficiency causes increases in inflammatory phenotypes at baseline.

**Figure 1:**
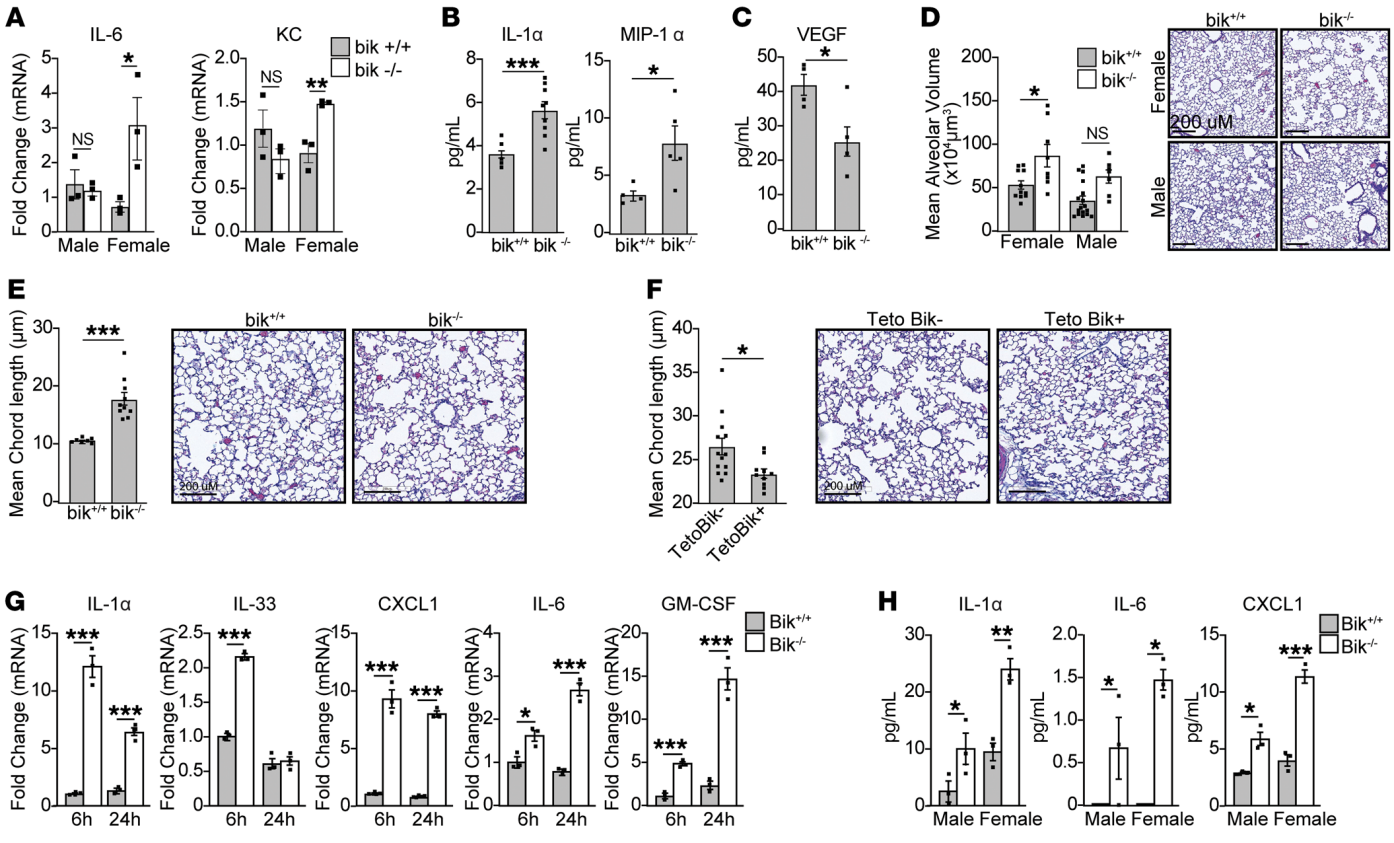
Loss of Bik in mice causes the development of emphysema. (**A**) Total RNA or protein was isolated from the cranial lobes of the lungs of 4-week-old mice, and changes in the basal expression levels of IL-6 and KC were analyzed by quantitative reverse-transcription PCR (qRT-PCR). *n* = 3/group. (**B**) The levels of IL-1α, MIP-1α, and (**C**) VEGF were analyzed in the lung homogenates of WT and *bik^–/–^* mice at 80 weeks of age by Luminex. *n* = 9/group. (**D**) Baseline weighted mean alveolar volume in *bik^+/+^* and old *bik^–/–^* mice at 13 to 44 weeks of age. *n* = 8–11; *N* = 2 (*n*, sample size in a single experiment; *N*, number of experimental repeats). (**E**) Mean alveolar chord length in 4-week-old female *bik^+/+^* and *bik^/^* mice. *n* = 3, *N* = 3. (**F**) Female CCSP-rtTA Bik transgenic mice and their littermates were kept with Dox water (400 mg/l) until 25 weeks of age. *n* = 10–13; *N* = 3. Mean alveolar chord length of lung tissues was measured in 10 randomly selected lung fields per mouse, using Visiopharm software. (**G**) MAECs isolated from female *bik^+/+^* and *bik^–/–^* mice were plated on 6-well plates. Media were changed to rock inhibitor-/FBS-free media and then cells harvested 6 and 24 hours later for mRNA analysis. The mRNA expression levels of the indicated inflammatory cytokines were analyzed using qRT-PCR from *n* = 3 wells/group. (**H**) Media from *bik^+/+^* and *bik^–/–^* MAECs plated on 6-well plates were analyzed for levels of IL-1α, IL-6, and KC using Luminex. *n* = 3 wells/group. The sample size in a single experiment is denoted as *n*; the number of experimental replicates is denoted as *N*. Two-tailed Student’s *t* test was used to compare between 2 groups, and grouped results were analyzed using 2-way ANOVA. Data are represented as mean ± SEM. *P < 0.05; **P < 0.01; ***P < 0.001.

*Bik^–/–^* mice breed normally and show no grossly different phenotypes compared with *bik^+/+^* mice as they age, and loss of Bik does not protect hematopoietic cells from cell death induced by a variety of stimuli (25). We found that Bik, as one of the Bcl-2 family of proteins, is crucial in the resolution of mucous cell hyperplasia in airway epithelia (20, 21, 24). However, we also observed that *bik^–/–^* compared with *bik^+/+^* mice displayed increased IL-6 and CXCL1 (KC) mRNA levels in lung homogenates, specifically in females (Figure 1A). Differences in lung homogenates were substantial only in female but not male *bik^–/–^* mice compared with *bik^+/+^* mice. To determine whether inflammation due to Bik deficiency increases as lungs age, we analyzed the levels of inflammatory cytokines in the lungs of mice at 80 weeks of age and detected higher levels of IL-1α and MIP-1α in *bik^–/–^* compared with *bik^+/+^* mice (Figure 1B). Further, *bik^–/–^* compared with *bik^+/+^* mice showed reduced VEGF levels in lung tissues (Figure 1C). Because reduced VEGF levels are associated with the development of emphysema (26), we quantified the alveolar volume to determine whether mice may present with emphysema as they get older. Because emphysema develops in aged mice, we initially analyzed the lungs of *bik^–/–^* and *bik^+/+^* mice at 56 to 80 weeks and found that the alveolar volume increased in the lungs of *bik^–/–^* compared with *bik^+/+^* mice at 56 to 80 weeks of age (Supplemental Figure 1A; supplemental material available online with this article; https://doi.org/10.1172/JCI170594DS1). We further noticed that emphysema was already evident in *bik^–/–^* but not in *bik^+/+^* mice at 13 to 44 weeks of age (Figure 1D). Interestingly, these differences were seen in the lungs of female mice, but not male mice (Figure 1D). Later, we noticed that emphysema was already present in female mice even when mice were 4 weeks old (Figure 1E).

We further explored whether inflammation in small airways of *bik^–/–^* mice may be responsible for emphysema development. This possibility was initially postulated from CT scans based on loss of terminal bronchioles that preceded emphysematous destruction and the association of functional small airway disease and emphysema and forced expiratory volume in 1 second (FEV_1_) decline (27–29). To determine whether Bik reduces inflammation in vivo when expressed only in the AECs, transgenic mice that conditionally induce Bik expression using the reverse tetracycline transactivator (rtTA) under the control of the Clara cell secretory protein (CCSP) promoter were generated and bred into the *bik*^–/–^ mouse. When these mice were placed on a doxycycline (Dox) diet for 48 hours, Bik expression was induced in the airways of CCSP-rtTA/TetOBik mice but not in CCSP-rtTA–only littermates (Supplemental Figure 1B). Therefore, CCSP-rtTA^+^TetOBik^−^ and CCSP-rtTA^+^TetOBik^+^ mice that are on the *bik^–/–^* background were kept with Dox-containing water until the age of 25 weeks to investigate whether inducible expression of Bik in the airways can mitigate the development of emphysema. Expression of Bik in the airways inhibited the development of emphysema in the lungs of CCSP-rtTA^+^TetOBik^+^ mice, while emphysema was still evident in the transgene-negative female mice (Figure 1F). However, male TetOBik^−^ and TetOBik^+^ mice showed no difference in the resolution of emphysema following Dox treatment until the age of 25 weeks (Supplemental Figure 1C).

### Baseline inflammation is elevated by bik deficiency.

Because our findings suggested that Bik has an antiinflammatory role, we investigated whether Bik-deficient airway cells also show inflammation at baseline. Bik mRNA was detected in *bik^+/+^* but not *bik^–/–^* mouse AECs (MAECs) (Supplemental Figure 1D), and *bik^–/–^* compared with *bik^+/+^* MAECs expressed increased IL-1α, IL-33, CXCL1, IL-6, and GM-CSF mRNA levels when cultured for 6 or 24 hours (Figure 1G). This finding was corroborated by detecting proinflammatory cytokines in the culture media using the Luminex system, as IL-1α, IL-6, and CXCL1 levels were increased in *bik^–/–^* compared with *bik^+/+^* MAECs (Figure 1H). Interestingly, the differences in inflammatory cytokine levels shown in Figure 1H were more pronounced in female mice. Primers used for PCR are listed in Supplemental Table 1.

### SNP rs738276 affects BIK promoter activity and expression.

**Figure 2:**
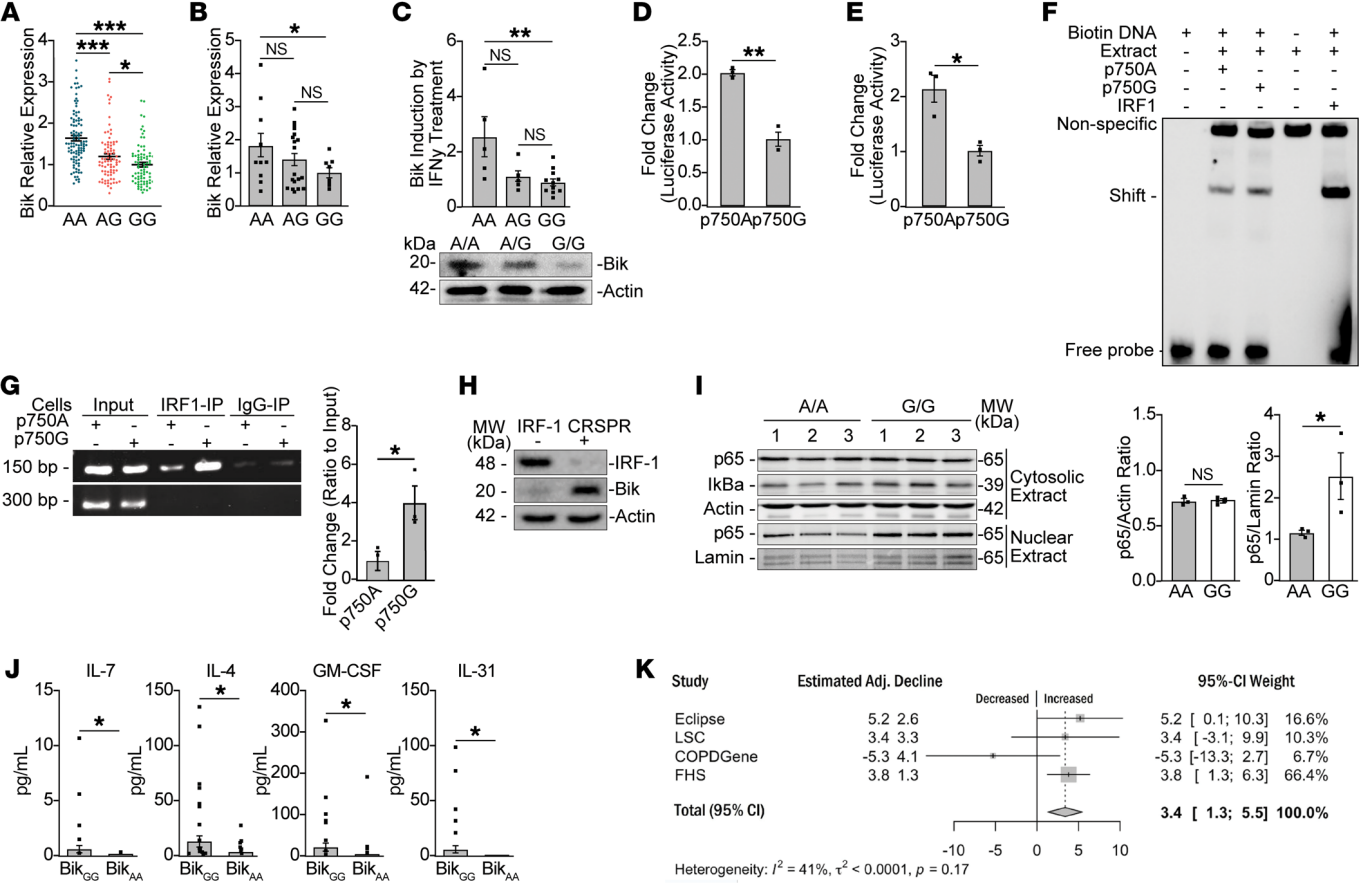
A SNP affects *BIK* promoter activity and expression (**A**) AA of rs738276 demonstrates higher expression than GG (*P* = 2 × 10^–11^) in lymphoblastoid cells. (**B**) *Bik* mRNA in 40 HAECs from current or former smokers (11 AA, 20 AG, 9 GG). (**C**) HAECs (*n* = 23) differentiated and treated with 50 ng/ml IFN-γ. Bik was increased in Bik AA compared with Bik GG HAECs (*P* = 0.002). Western blot analysis of Bik protein in Bik AA and Bik GG HAECs. (**D** and **E**) p750A-SA or p750G-SA constructs were transfected into (**D**) HAECs and (**E**) H1299 cells, and *BIK* gene promoter activity of A normalized to G values. *n* = 3; *N* = 3. (**F**) IRF-1 has a higher affinity to p750G compared with p750A. Nuclear extracts from H1299 cells were subjected to EMSA using biotin-labeled p750A or p750G oligonucleotide probes of Bik and IRF-1 genes. (**G**) Primary HAECs with Bik AA and Bik GG genotypes were processed for ChIP assays by using anti–IRF-1 or control IgG (negative control). Precipitated DNA was subjected to PCR to amplify the Bik gene fragment. (**H**) Protein lysates from WT and IRF-CRISPR knockout HAECs subjected to Western blotting. (**I**) Cytosolic and nuclear fractions of BikAA and BikGG HAECs analyzed for expression of indicated proteins by Western blotting. (**J**) Plasma samples from individuals with Bik AA and Bik GG genotypes analyzed using Luminex. In each group, 40 samples were analyzed, and in many samples, these cytokines were not detected. (**K**) Metaanalysis of increased decline in lung function associated with G allele in study participants of more than 60 years of age in ECLIPSE, LSC, COPDGene, and FHS. Additive model compared GG, GA, and AA genotypes of *BIK* SNP rs738276 using GG as a reference group. Two-tailed Student’s *t* test compared between 2 groups, and grouped results were analyzed using 2-way ANOVA. Data are represented as mean ± SEM. *P < 0.05; **P < 0.01; ***P < 0.001.

Based on a putative promoter region located from the UCSC Genome Browser (https://genome.ucsc.edu/), rs738276 is in the first intronic region of *BIK*, where transcription binding sites are enriched (Supplemental Figure 2A). Therefore, we evaluated *BIK* expression using publicly available microarray data for lymphoblastoid cell RNA from HapMap participants (30), and individuals with the AA compared with the GG genotype demonstrated higher Bik mRNA levels, with intermediate levels in cells with the AG genotype (Figure 2A). African, European, or Asian ancestry did not affect Bik expression levels (Supplemental Figure 2B). Further, smoking status of donors (current versus former) did not influence the effect of genotype on Bik expression (Supplemental Figure 2C). We confirmed the findings in lymphoblastoid cells in primary HAECs from 11 subjects with AA compared with 9 subjects with GG genotypes that showed 1.82-fold higher Bik mRNA levels, while HAECs from 20 subjects with the AG genotype displayed intermediate levels (*P* = 0.03, AA versus GG) (Figure 2B). Because we have previously shown that IFN-γ induces Bik expression in HAECs (20), we investigated whether this genotype enhances the effect of IFN-γ on Bik expression (20). We found that IFN-γ treatment of differentiated cultures induced even higher Bik mRNA levels in HAECs with the AA compared with the GG genotype (Figure 2C). Not only Bik mRNA, but also protein levels, were higher in HAECs with the AA compared with GG genotype (Figure 2C).

To further understand the role of this SNP, we generated a promoter luciferase construct, p750A-SA, that was active when a splice-acceptor site was included (Supplemental Figure 2D), and the activity was highest in H1299 cells, although also detectable in other cell lines, including AALEB, N1, and NIH-292 cells (Supplemental Figure 2E). The promoter construct p750A-SA compared with p750G-SA showed approximately 1.8-fold higher luciferase activity in the AALEB AEC line (Figure 2D), and similar findings were observed in H1299 cells (Figure 2E). The sequence encompassing rs738276 showed a high homology to the consensus IRF-1–binding site. Therefore, EMSAs were carried out using biotin-labeled double-stranded oligonucleotides, and we found that oligoA or oligoG was bound to a protein similar to the consensus IRF-1–binding oligonucleotide (IRF-1oligo) when incubated with nuclear extracts. Sequences of primers used for EMSA and ChIP assays are listed in Supplemental Table 2. OligoG compared with oligoA showed stronger shifted band, and oligoIRF-1 displayed the strongest band (Figure 2F). Binding specificity was confirmed by competition with excess nonlabeled oligoA and oligoG oligonucleotides (Supplemental Figure 2F) and with nonlabeled IRF-1oligo (Supplemental Figure 2G). Further, differential interaction was shown by chromosome immunoprecipitation assays with anti–IRF-1 antibodies pulling down 4-fold more DNA from HAEC homozygotes for the GG compared with the AA genotypes (Figure 2G). In AECs, suppression of IRF-1 using CRISPR constructs increased endogenous Bik expression in HAECs (Figure 2H), suggesting transcriptional factor IRF-1 negatively regulates Bik expression. Collectively, these data show that IRF-1 preferably binds to the *BIK* intronic region with the G rather than the A allele at the rs738276 locus.

We next determined whether genotype-dependent reduction in Bik protein affects nuclear p65 levels, as p65 is a central transcription factor for many inflammatory mediators (31). While the cytosolic p65 and IκBα levels were not affected, nuclear p65 levels were increased in primary HAECs with the GG compared with the AA allele (Figure 2I). Further, HAECs with the GG compared with the AA allele when treated with TNF-α showed increased IL-6 and IL-8 mRNA levels (Supplemental Figure 2H).

### Reduced Bik levels cause increased proinflammatory phenotype in humans.

Comparison of plasma samples from 40 individuals, each with the AA and GG alleles of the BIK SNP rs738276, that were matched for sex and age (Table 1) showed that circulating IL-7, IL-4, GM-CSF, and IL-1 levels were higher in plasma of people with the GG than people with the AA allele (Figure 2J). IL-5, IL-8, IL-12, IL-13, IL-18, MCP1α, MCP1β, and TNF-α showed no differences between groups (data not shown).

### Association of SNP rs738276 with decline in lung function in humans.

We identified 4 cohorts of cigarette smokers for whom longitudinal lung-function measurements were available. The non-Hispanic white participants for the Lovelace Smokers Cohort (LSC) (32), Framingham Heart Study (FHS) (33, 34), Evaluation of COPD Longitudinally to Identify Predictive Surrogate Endpoints (ECLIPSE) (35), and COPDGene Study (36) were 74.4%, 53.3%, 30.6%, and 46.7% female, respectively (Table 2). In ECLIPSE, all selected participants showed chronic airflow obstruction (CAO) as defined by FEV_1_/forced vital capacity (FVC) ≤ 0.7 and FEV_1_ percent predicted <80%. Overall, participants in the 4 cohorts had a mean FEV_1_ decline that ranged from –29.4 to –42.5 mL/year. The median length of follow-up was at least 3 years and up to 10 years in the COPDGene study. After adjusting for sex, BMI, smoking pack years, and smoking status, none of the cohorts showed a statistically significant association with decline in lung function when all participants or participants younger than 60 years of age were used for analyses. However, for participants 60 years of age and older, meta-analysis results showed a significant association (Figure 2K). In an additive model, each additional copy of the G allele in individuals more than 60 years of age led to a 3.4 ml/year excess FEV_1_ decline (95%CI = [1.3, 5.5]) by random effect model with moderate heterogeneity (I^2^ = 41%). Within the ECLIPSE, LSC, and FHS, each copy of the G variant that led to reduced *BIK* expression was associated with excess decline of 5.2, 3.4, and 3.8 ml/year, respectively. While the COPDGene was the largest study, it had a lower number of visits and longer time between visits, and measurements were obtained over a longer period with a higher number of deaths during this period. We were not able to see marked differences between genotypes when the analyses were stratified by sex, likely because of insufficient sample size and lack of statistical power. Further, this association with decline in lung function was not observed in the Normative Aging Study cohort (37), which represented male study participants only, who on average were 41 years of age.

### Bik deficiency enhances allergen- and LPS-induced inflammation in the lungs.

**Figure 3:**
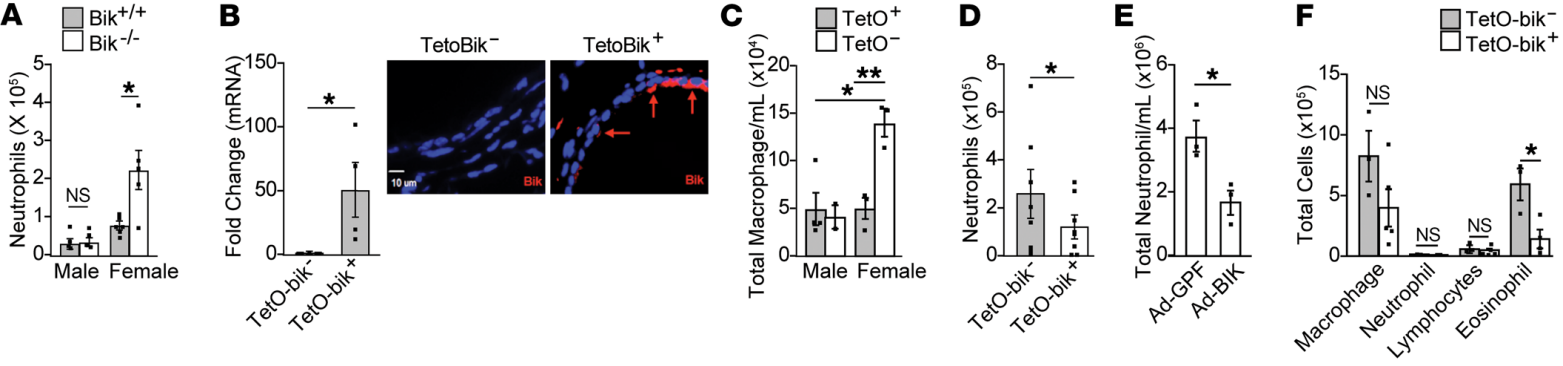
Bik suppresses LPS and allergen-induced inflammation in mice. (**A**) *bik^+/+^* and *bik^–/–^* mice were instilled with 5 μg LPS intranasally, and neutrophil numbers were compared in the BAL fluid 4 hours later. *n* = 5/group. (**B**) TetoBik^–^ and tetoBik^+^ mice were put on Dox diet for 48 hours, and the expression of Bik was analyzed in the lung tissues by qRT-PCR or immunostaining. Bik mRNA and protein levels were shown by qRT-PCR and immunostaining, respectively. (**C**) Baseline macrophage levels in the BAL fluid of male and female CCSP-rtTA^+^TetOBik^+^ and CCSP-rtTA^+^TetOBik^–^ mice, 2 male and 3 female in TetOBik^–^ and 4 male and 3 female in CCSP-TetOBik^–^ per group. (**D**) TetoBik^–^ and TetoBik^+^ mice were placed on Dox diet for 48 hours and subsequently instilled with 50 μg LPS and lungs harvested; BAL fluid neutrophil numbers were quantified 4 hours later (*n* = 7/group 4 males and 3 female). (**E**) Female *bik^–/–^* mice were instilled once daily over 2 days with 10^8^ virus particles of Ad-Bik or Ad-GFP, as control, diluted in final volume of 50 μL in PBS. Twenty-four hours later, mice were intranasally instilled with 5 μg LPS and euthanized and BAL fluid neutrophil numbers quantified 24 hours later. *n* = 4/group. (**F**) TetoBik^–^ and TetoBik^+^ mice were placed on Dox diet for 48 hours and subsequently instilled with 10 μg HDM for 5 days (*n* = 5/group). BAL cell numbers were analyzed 5 days later. Two-tailed Student’s *t* test was used to compare between 2 groups, and grouped results were analyzed using 2-way ANOVA. Data are represented as mean ± SEM. *P < 0.05; **P < 0.01.

As Bik-deficient mice caused increased inflammation at baseline, we investigated whether Bik may protect from inflammation when mice are challenged with inducers of neutrophilic or eosinophilic inflammation. We observed that 4 hours after instillation with 5 μg LPS, neutrophil numbers were significantly increased in the bronchoalveolar (BAL) fluid of female *bik^–/–^* compared with *bik^+/+^* mice (Supplemental Figure 3A and Figure 3A). However, to determine whether Bik may protect from extensive inflammation, we instilled mice with 50 μg LPS and found that at that dose, Bik deficiency caused enhanced inflammation in *bik^–/–^* compared with *bik^+/+^* mice, irrespective of sex (Supplemental Figure 3B).

To determine whether Bik reduces inflammation in vivo when expressed only in the AECs, transgenic mice that conditionally induce Bik expression using rtTA under the control of the CCSP promoter were used. When these mice were placed on a Dox diet for 48 hours, Bik expression (Supplemental Figure 3C) was induced in the airways of CCSP-rtTA^+^TetOBik^+^ mice, but not in CCSP-rtTA^+^TetOBik^–^ littermates (Figure 3B). At baseline, while the male CCS-rtTA^+^TetOBik^+^ and CCSP-rtTA^+^TetOBik^–^ mice showed no difference in the number of cells in the BAL, the female TetOBik^–^ compared with TetOBik^+^ mice showed a 3-fold increase in the number of macrophages (Figure 3C). No differences were observed in the numbers of neutrophils or lymphocytes (data not shown). When these mice were instilled with LPS, the number of neutrophils was reduced by Bik expression when Bik expression was induced in airways of mice and intranasally instilled with LPS (Figure 3D). At 24 hours after mice were instilled with 5 μg LPS, inflammation was not different between *bik^–/–^* and *bik^+/+^* mice (Supplemental Figure 3D), but mice that received an adenoviral expression vector for Bik (Ad-Bik) compared with Ad-GFP showed reduced neutrophils (Figure 3E). These findings show that, while the endogenous Bik suppressed inflammation at early stages, endogenous Bik was no longer effective in reducing inflammation 24 hours after LPS instillation; however, increased expression of Bik was efficient in also reducing inflammation at the 24-hour time point. To determine whether Bik expression also suppressed inflammation in a mouse model of asthma, we sensitized mice with house dust mite (HDM) allergen by 2 different methods, as described (38). The numbers of BAL eosinophils were reduced in CCSP-rtTA/TetOBik compared with CCSP-rtTA^+^TetOBik controls exposed to HDM allergen for 5 consecutive days over 4 weeks (Figure 3F and Supplemental Figure 3E). Bik expression also suppressed inflammation in a modified mouse model of asthma, where mice were sensitized with HDM via intranasal instillation on days 1 and 8 and subsequently challenged with HDM 5 d/wk for 4 weeks and kept on a Dox diet during this time. The number of eosinophils was reduced in the BAL fluid of CCSP-rtTA/TetOBik compared with CCSP-rtTA^+^TetOBik controls (Supplemental Figure 3F).

### The BH3 domain of Bik inhibits nuclear p65–induced transcriptional activity.

**Figure 4:**
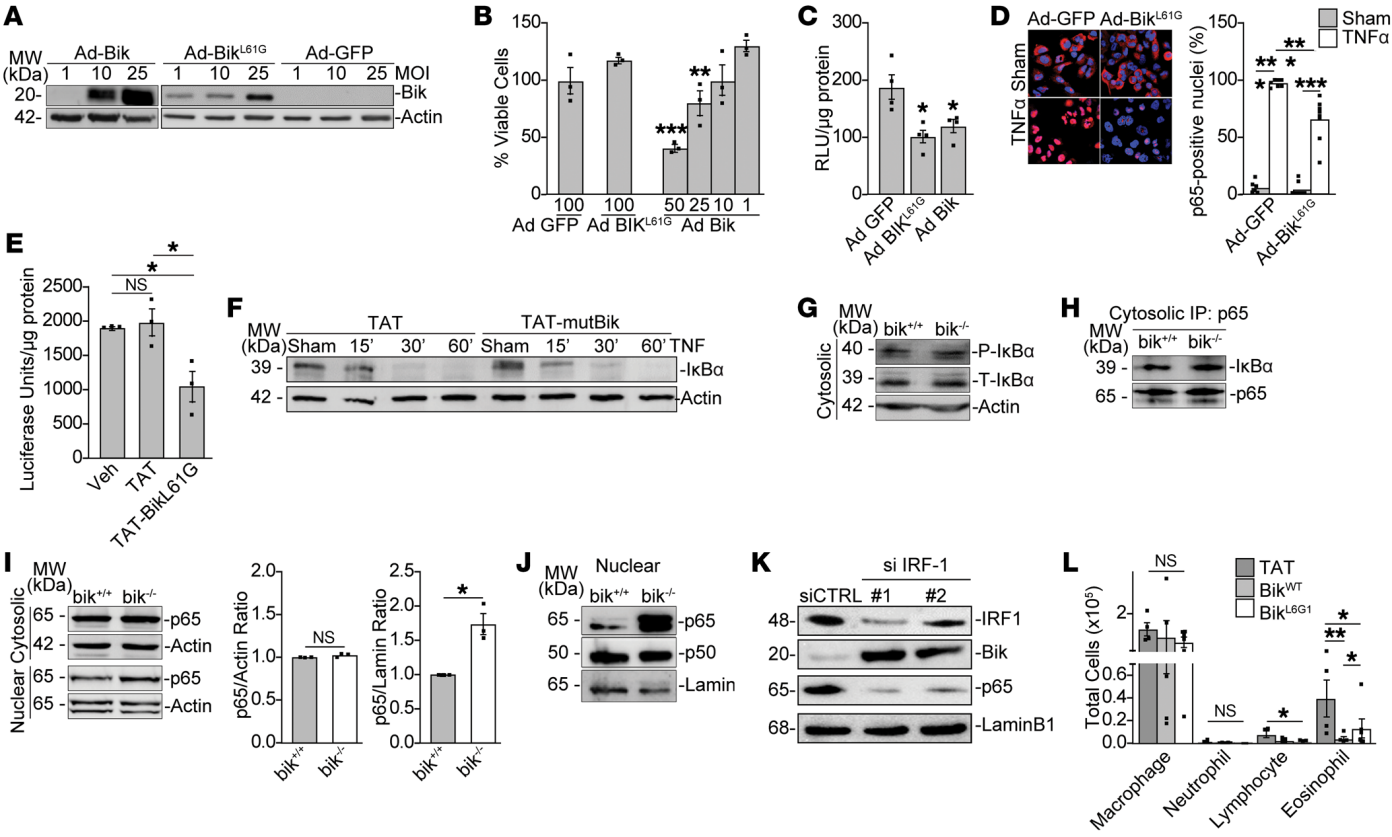
The BH3 domain of Bik inhibits nuclear p65–induced transcriptional activity. A549 NF-κB luciferase reporter cells were infected with either Ad GFP, Ad-Bik, or Ad-Bik^L61G^ (mutant Bik that does not kill cells) and subsequently treated with 10 ng/ml TNF-α for 6 hours. (**A**) Protein lysates were analyzed for the expression of Bik by Western blotting. (**B**) Percentages of viable cells were analyzed by trypan blue exclusion assay. *n* = 3. Cells were infected with the adenoviral vectors at MOIs indicated below the bar. (**C**) NF-κB transcriptional activity was analyzed in the cell lysates. *n* = 4. (**D**) Percentages of cells expressing nuclear p65 were analyzed by immunofluorescent staining. *n* = 6–8; *N* = 2. (**E**) A549 NF-κB luciferase reporter cells were treated with vehicle, TAT, or TAT-Bik^L61G^ peptides for 2 hours and subsequently treated with TNF-α for 6 hours. NF-κB transcriptional activity was analyzed in the cell lysates. *n* = 3. (**F**) A549 cells were treated with 10 μM control peptides or Bik^L61G^ peptides for 2 hours, followed by treatments with 10 ng/ml TNF-α for the indicated times in the presence of phosphormide. Cell lysates were analyzed for the level of IκBα protein by Western blotting. (**G**) Cytosolic-protein lysates from *bik^+/+^* and *bik^–/–^* MAECs were analyzed for levels of phospho- and total IκBα by Western blotting. (**H**) Cytosolic lysates from *bik^+/+^* and *bik^–/–^* MAECs were immunoprecipitated using anti-p65 antibody and analyzed for IκBα levels by Western blotting. (**I**) Cytosolic and nuclear fractions of lysates from *bik^+/+^* and *bik^–/–^* MAECs were analyzed for levels of p65 and IκBα by Western blotting. (**J**) Nuclear fractions of lysates isolated from *bik^+/+^* and *bik^–/–^* MAECs analyzed for levels of p65 and p50 by Western blotting. (**K**) HAECs transfected with siControl or IRF-1 siRNA. Western blot of nuclear lysates for Bik and p65 protein. (**L**) *bik^–/–^* mice instilled with 50 μg HDM intranasally daily for 5 consecutive days and on days 6 and 7 intranasally treated with 10 μM of control TAT peptide, BH3 WT Bik peptide, or BH3 mutant Bik peptide. BAL fluids analyzed for inflammatory cell numbers. *n* = 4–6/group. Two-tailed Student’s *t* test was used to compare between 2 groups, and grouped results were analyzed using 2-way ANOVA. Data are represented as mean ± SEM. *P < 0.05; **P < 0.01; ***P < 0.001.

Our earlier studies have demonstrated that a mutation of Bik within the BH3 domain (Leu61Gly) nullifies the apoptotic activity in AECs (20, 24). To investigate the underlying mechanisms and whether the proapoptotic activity of Bik is required for its antiinflammatory action, A549 cells stably transfected with a luciferase reporter construct that is driven by multiple copies of the NF-κB response element (A549-NF-κB-luc cell line) were infected with either Ad-GFP, Ad-Bik^WT^, or the mutant Ad-Bik^L61G^ and expression of Bik was determined (Figure 4A). Mutation of L61G in the BH3 domain of Bik nullifies its apoptotic activity. Bik^WT^ but not Bik^L61G^ interacts with and suppresses nuclear translocation of phospho-ERK1/2, and suppression of ERK1/2 activation inhibits IFN-γ– and Bik-induced cell death (20). Furthermore, expression of Bik^WT^ but not Bik^L61G^ dissociated Bcl-2 from BAK to initiate cell death in the AECs (24). As in previous studies, AdBik^WT^ reduced cell viability in a dose-dependent manner, while Ad-Bik^L61G^ and Ad-GFP did not cause cell death (Figure 4B). When cells were treated with TNF-α for 6 hours, both Ad-Bik^WT^ and Ad-Bik^L61G^ equally suppressed TNF-α–induced NF-κB transcriptional activity compared with Ad-GFP controls (Figure 4C). Immunofluorescence confirmed that nuclear p65 positivity was diminished by the mutant Ad-Bik^L61G^ compared with Ad-GFP (Figure 4D). Similarly, treatment of cells with the mutant Bik-derived BH3 peptide TAT-Bik^L61G^ dampened NF-κB activation (Figure 4E). However, IκBα degradation (Figure 4F), which allows NF-κB to translocate to the nucleus (39, 40), was not affected by the Bik peptide. Because the in vivo studies showed that inflammation was pronounced in female mice, the in vitro studies used MAECs that were obtained from female mice. The levels of IκBα (Figure 4G) and p65/IκBα interactions (Figure 4H) in the cytosolic fractions showed no differences between *bik^+/+^* and *bik^–/–^* MAECs. Levels of nuclear p65 were consistently increased in the *bik^–/–^* MAECs (Figure 4, I and J), whereas nuclear p50 levels was not affected by *Bik* deficiency (Figure 4J). These data show that the proinflammatory effect of Bik affects nuclear p65 levels without enhancing degradation of IκBα. Further, as IRF-1 was involved in regulating Bik expression, we investigated to determine whether suppressing IRF-1 would also modify nuclear p65 levels. Suppression of IRF-1 using siRNA constructs resulted in increased Bik expression and reduced nuclear p65 levels (Figure 4K).

NF-κB is a proinflammatory transcription factor that regulates inflammation in the lung tissues (11, 41, 42). In the absence of TNF-α treatment, substantial increases in p65 levels were observed in the nuclear extract of *bik^–/–^* compared with *bik^+/+^* MAECs at baseline. However, these differences were no longer present when cells were stimulated with increasing doses of TNF-α (Supplemental Figure 4A), suggesting that large amounts of p65 that will be translocated into the nucleus by TNF-α treatment overwhelm the initially Bik-mediated reduction of nuclear p65. Consistent with these observations, intranasal instillation of mice with TAT-Bik^L61G^ peptide on 2 consecutive days after 5 days of HDM exposure (Supplemental Figure 4B) reduced HDM-induced lymphocytic and eosinophilic inflammation in the lungs of *bik*-deficient mice (Figure 4L). Collectively, these results demonstrate that the BH3 domain of Bik inhibits NF-κB–mediated inflammation by mechanisms different from those of the cell death–inducing activity, as the nonapoptotic mutant Bik^L61G^ peptide also dampens inflammation in *bik*-deficient mice.

### Bik/Bcl-2 complex causes degradation of nuclear p65.

**Figure 5:**
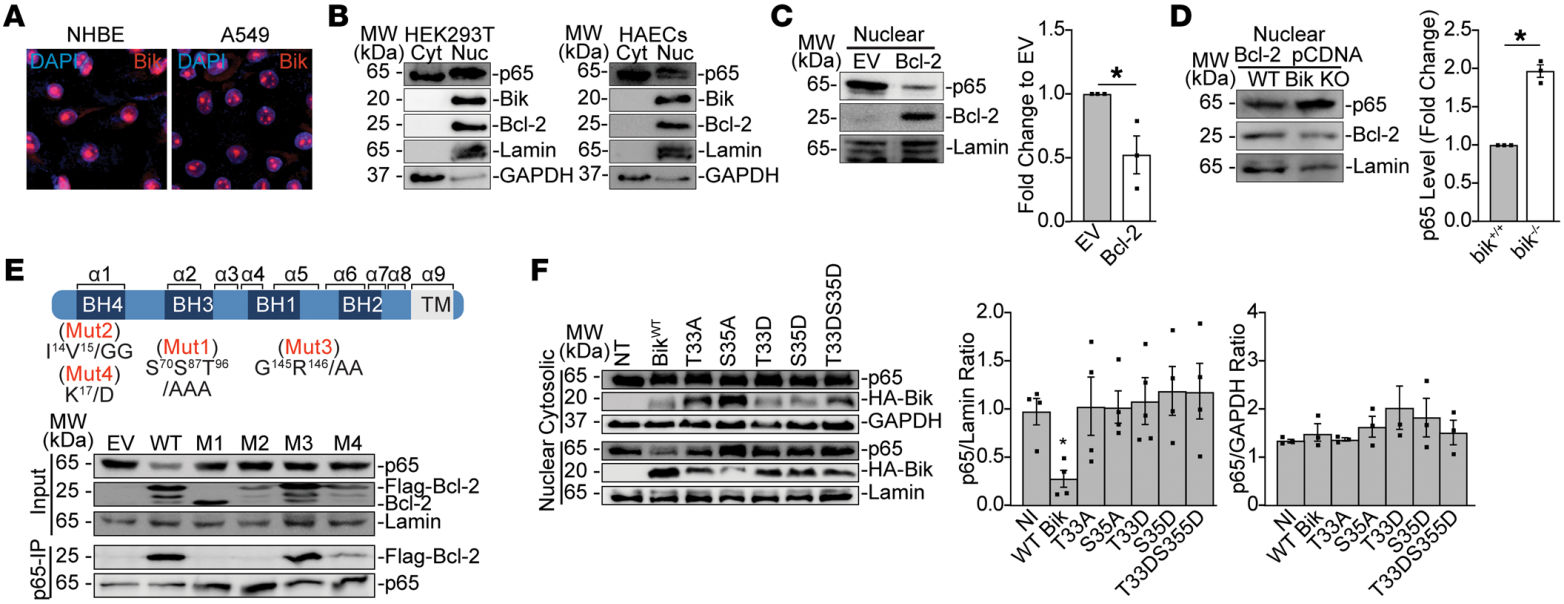
Bik, localized to nuclear domains of the cell, reduces nuclear p65 by binding to Bcl-2. (**A**) Primary HAECs and A549 cells were immunostained for Bik protein and analyzed by fluorescent microscopy for the localization of Bik protein. Original magnification, ×1,200. (**B**) Cytosolic and nuclear extracts from HEK293T and HAECs were analyzed for the localization of p65, Bik, and Bcl-2 by Western blotting. (**C**) HEK293T cells were transfected with EV or Bcl-2 plasmid, and protein lysates were analyzed for Bcl-2 and p65 levels by Western blotting. (**D**) WT and CRISPR/cas9 Bik-knockout HEK293T cells were transfected with plasmids expressing WT Bcl-2. Forty-eight hours later, nuclear lysates were analyzed for levels of p65 and Bcl-2 by Western blotting. The bar graph on the right-hand side shows densitometry (p65 fold change) of the Western blot from 3 independent experiments. (**E**) CRISPR/Cas9 Bcl-2–knockout cells were transfected with EV, WT, or mutant triple–flag-tagged Bcl-2. Forty-eight hours later, nuclear lysates were analyzed for the expression levels of p65 using Western blotting, nuclear lysates were immunoprecipitated using anti-p65 or Bcl-2 antibodies, and the Ips were probed for flag–Bcl-2 and p65. (**F**) Bik CRISPR/Cas9 knockout cells were transfected with EV or plasmids expressing WT or phosphorylation-mutant Bik constructs. Forty-eight hours later, cytosolic and nuclear lysates were analyzed for p65 levels by Western blotting. Two-tailed Student’s *t* test was used to compare between 2 groups, and grouped results were analyzed using 2-way ANOVA. Data are represented as mean ± SEM. *P < 0.05.

To begin elucidating the mechanisms by which Bik reduced nuclear p65 levels, we first investigated the cellular localization of Bik. Bik when increased in expression localizes to the ER (43); however, immunostaining of A549 and primary HAECs at baseline demonstrated that Bik was primarily localized to the nucleus (Figure 5A), which was confirmed by immunoblotting of cytosolic and nuclear fractions of HEK293T cells and HAECs, where both Bik and Bcl-2 were detected in the nuclear fractions (Figure 5B). Cytosolic, perinuclear, and nuclear proteins were prepared from *bik^+/+^* and *bik^–/–^* MAECs using increasing Igepal and NaCl_2_ concentrations. Large amounts of Bcl-2 were detected in the perinuclear fraction where the light and heavy membranes are found, as shown by the mitochondrial and ER markers Tom20 and calnexin (Supplemental Figure 5A). However, substantial amounts of Bcl-2 were detected also in the nuclear fraction (Supplemental Figure 5A).

Because Bik binds Bcl-2 via its BH3 domain (24), we examined the role of Bcl-2 in reducing p65. We expressed WT Bcl-2 in HEK293T cells and isolated the nuclear fraction after removing the cytosolic fraction and the perinuclear fraction, i.e., including the ER and mitochondria. We found that WT Bcl-2–expressing cells drastically reduced nuclear p65 levels (Figure 5C). Furthermore, we found that the ER-targeted Bcl-2 also degraded nuclear p65 (Supplemental Figure 5B) and interacted with p65 (Supplemental Figure 5, B and C). Bik has a high affinity to Bcl-2 and dissociates Bcl-2 from Bak and the inositol trisphosphate receptor (IP_3_R) in the cytosol to induce cell death (24). Therefore, we postulated that Bik may affect Bcl-2 in the nucleus to modify p65 levels. Bik-deficient HEK293T cells that were generated by targeting Bik using CRISPR/Cas9 and expanded from clone no. 7 (Supplemental Figure 5D) showed increased accumulation of nuclear p65 protein levels upon expression of Bcl-2 (Figure 5D). To identify the domains within the Bcl-2 protein that affect p65 levels, we generated Bcl-2 knockout HEK293T cells using CRISPR/Cas9 (Supplemental Figure 5E) and transfected cells expanded from clone no. 4 with either empty vector (EV) as control or constructs with triple–flag-tagged WT, BH1-, BH4-, or triple phospho-mutant Bcl-2 (Figure 5E). While the WT Bcl-2 caused degradation of nuclear p65, the BH1, BH4, or the triple-phosphorylation mutants did not affect the levels of nuclear p65 (Figure 5E). However, immunoprecipitation with anti-p65 antibodies showed that WT Bcl-2 and the BH1 mutant interacted with nuclear p65, while mutations in the BH4 domain impaired Bcl-2/p65 interactions (Figure 5E). The phosphorylation mutant Bcl-2 also showed no interaction with nuclear p65.

We have reported that phosphorylation of Bik at T33 and S35 is required for Bik to cause cell death in epithelial cells in a cell cycle–dependent manner (44). Thus, we explored the role of Bik phosphorylation in facilitating p65 degradation using Bik expression constructs that have either constitutively nonphosphorylated (T33A and S35A) or constitutively phosphorylated (T33D, S35D, and T33/S35DD) mutants. We observed that nonphosphorylatable Bik alanine mutants primarily localize to the cytosol and did not enrich in the nuclei, while the phosphorylation-mimicking Bik aspartate mutants were minimally detected in the cytosol but shuttled to the nucleus. However, unlike the WT Bik, all Bik phospho-mutants regardless of phosphorylation status did not cause reduction of nuclear p65 levels (Figure 5F). These findings suggest that while phosphorylation of Bcl-2 may be playing a role, Bik phosphorylation did not play a role in Bik binding to nuclear Bcl-2 and in Bcl-2 interacting with nuclear p65.

### Bik cooperates with Bcl-2 to promote proteasomal degradation of p65.

**Figure 6:**
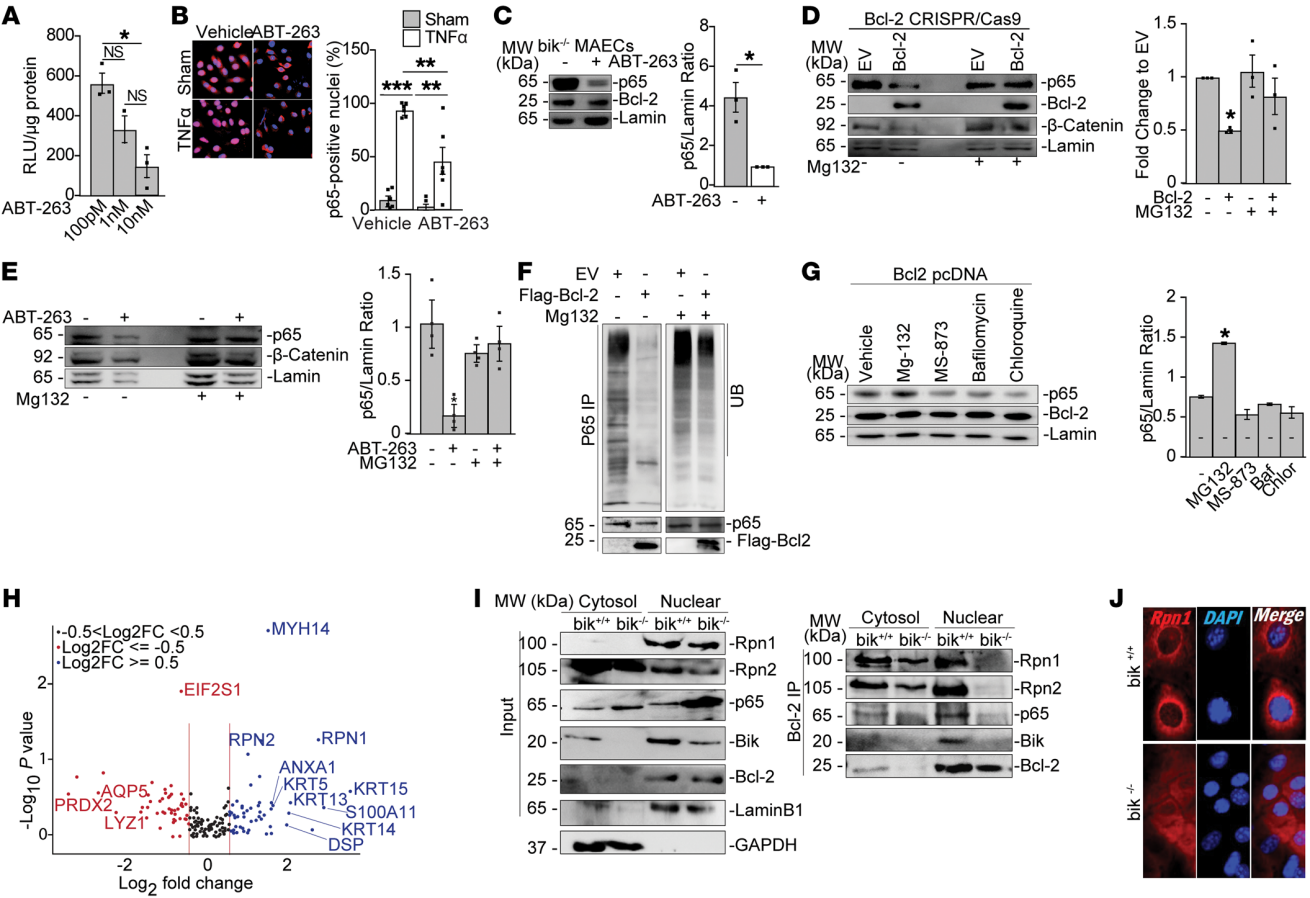
Bik through Bcl-2 interacts with the S19 regulatory particle components of the proteasome and regulates protein degradation. (**A**) A549 NF-κB luciferase reporter cells treated with ABT-263 and assessed for NF-κB transcriptional activity 24 hours later (*n* = 3). (**B**) A549 cells expressing NF-κB luciferase reporter treated with vehicle or 10 nM ABT-263 for 24 hours, followed by treatments with sham or 10 ng/ml TNF-α for 6 hours, and analyzed by immunostaining. *n* = 6; *N* = 2. (**C**) *bik^–/–^* MAECs treated with mock or 10 nM ABT-263 for 24 hours. Nuclear fractions of the cell lysates probed for p65 on Western blots. (**D**) Bcl-2 CRISPR/Cas9 knockout HEK293T cells transfected with 0.5 μg EV or Bcl-2 plasmids and 48 hours later treated with vehicle or 20 μM MG132 for 4 hours. Nuclear fractions probed for p65, β-catenin, and lamin. (**E**) *Bik*^–/–^ MAECs treated with mock or 10 nM ABT-263 for 24 hours followed by treatment with vehicle or 20 μM MG132 for 4 hours. Nuclear fraction probed for p65, β-catenin, and lamin A/C. (**F**) Bcl-2 CRISPR/Cas9 knockout HEK293T cells transfected with EV or flag–Bcl-2 and treated with vehicle or 20 μM MG132 for 4 hours. Nuclear lysates immunoprecipitated 4 hours later using anti-p65 antibody and the IPs analyzed for p65 ubiquitination using antiubiquitin. (**G**) Bcl-2 CRISPR/Cas9 knockout HEK293T cells transfected with Bcl-2 plasmid for 48 hours and treated with vehicle or 20 μM MG132, 10 μM MS-873, 10 μM bafilomycin, or 10 μM chloroquine for 4 hours. Nuclear lysates were analyzed for levels of p-65. (**H**) Volcano plot from Bcl-2 immunoprecipitates of nuclear lysates from *bik^+/+^* and *bik^–/–^* MAECs. Blue dots indicate proteins enriched in *bik^+/+^* MAECs while red dots indicate proteins increased in *bik^–/–^* MAECs; black dots indicate no change between *bik^+/+^* and *bik^–/–^* MAECs. (**I**) Cytosolic and nuclear fractions of *bik^+/+^* and *bik^–/–^* MAECs analyzed for p65, RPN1, RPN2, and Bik levels by Western blotting and immunoprecipitates using anti–Bcl-2 were analyzed for RPN1 and RPN2 levels by Western blotting. (**J**) *bik^+/+^* and *bik^–/–^* MAECs grown on cell-culture chambers and analyzed for localization of RPN1 by confocal microscopy. Two-tailed Student’s *t* test was used to compare between 2 groups, and grouped results were analyzed using 2-way ANOVA. Data are represented as mean ± SEM. *P < 0.05; **P < 0.01; ***P < 0.001. Original magnification, ×1,500 (**B**); ×500 (**J**).

To determine whether the BH3 groove of Bcl-2 plays a role in the Bik-mediated suppression of inflammation, we used the small molecule BH3 mimetic ABT-263 (Navitoclax), which mimics the binding of BH3 peptides to the hydrophobic BHdomain–binding groove and inhibits Bcl-2, Bcl-X_L_, and Bcl-w (45). When A549–NF-κB–luc cells were treated with different doses of ABT-263, NF-κB transcriptional activity was inhibited in a dose-dependent manner (Figure 6A). Also, TNF-α–induced nuclear p65 positivity was diminished in the nuclei of ABT-263–treated cells compared with controls (Figure 6B), suggesting that binding to the BH3-binding groove was required for Bcl-2 to inhibit NF-κB. Increased levels of p65 in *bik^–/–^* MAECs were reduced by treatment with ABT-263 (Figure 6C). Furthermore, expression of Bcl-2 in Bcl-2–deficient HEK293T cells caused degradation of nuclear p65, which was restored by MG132 treatment. Inhibition of proteasomal degradation was also shown by increased β-catenin levels in cells treated with MG132 (Figure 6D). These findings suggest that Bcl-2 facilitated proteasomal degradation of p65. Further, ABT-263–induced degradation of nuclear p65 in female *bik^–/–^* MAECs was also prevented by MG132 treatment (Figure 6E). Protein extracts from Bcl-2–deficient HEK293T cells that were transfected with EV or WT Bcl-2 plasmids and analyzed by immunoprecipitation using antibodies to p65 showed that Bcl-2 drastically reduced total ubiquitination in these cells and that this reduction in Bcl-2–mediated ubiquitination was partially rescued by MG132 (Figure 6F). While treatment with MG132 inhibited Bcl-2–induced degradation of p65, treatments with the ERAD inhibitor MS-873 or the lysosomal degradation inhibitors bafilomycin and chloroquine did not affect Bcl-2–induced degradation of p65 (Figure 6G), further supporting that nuclear Bcl-2 enhances proteasomal degradation of proteins.

To understand the role of Bcl-2 in proteasomal degradation, nuclear extract was prepared after removing the perinuclear proteins using increasing Igepal and NaCl_2_ concentrations. The nuclear extracts from female *bik^+/+^* and *bik^–/–^* MAECs were then subjected to immunoprecipitation using anti–Bcl-2 antibodies to identify the interacting proteins (Supplemental Figure 6A) and submitted for proteomic analyses. Volcano plots showed that, among the interacting proteins, the most represented proteins were RPN1, RPN2, keratins, and myosins (Figure 6H). These findings suggested that Bik/Bcl-2 interaction may be involved in the shuttling of proteasomes from the cytosolic to the nuclear compartments. Consistent with the proteomics data, our immunoprecipitation analyses showed that RPN1 and RPN2 interacted with both p65 and Bcl-2 in *bik^+/+^*, while this interaction was impaired in the nuclear extracts of *bik^–/–^* cells (Figure 6I). Consistent with these findings, when visualized by electron microscopy, the perinuclear region of *bik^–/–^* AECs was void of cellular organelles compared with those from *bik^+/+^* mice (Supplemental Figure 6B). To verify whether the nuclear localization of RPN1 is affected by the presence of Bik, we immunostained MAECs from *bik^+/+^* and *bik^–/–^* mice and found that RPN1 was localized in the perinuclear regions in *bik^+/+^* MAECs and diffusely distributed in the cytosol in *bik^–/–^* MAECs (Figure 6J).

### Bcl-2 levels are reduced in female airway cells.

**Figure 7:**
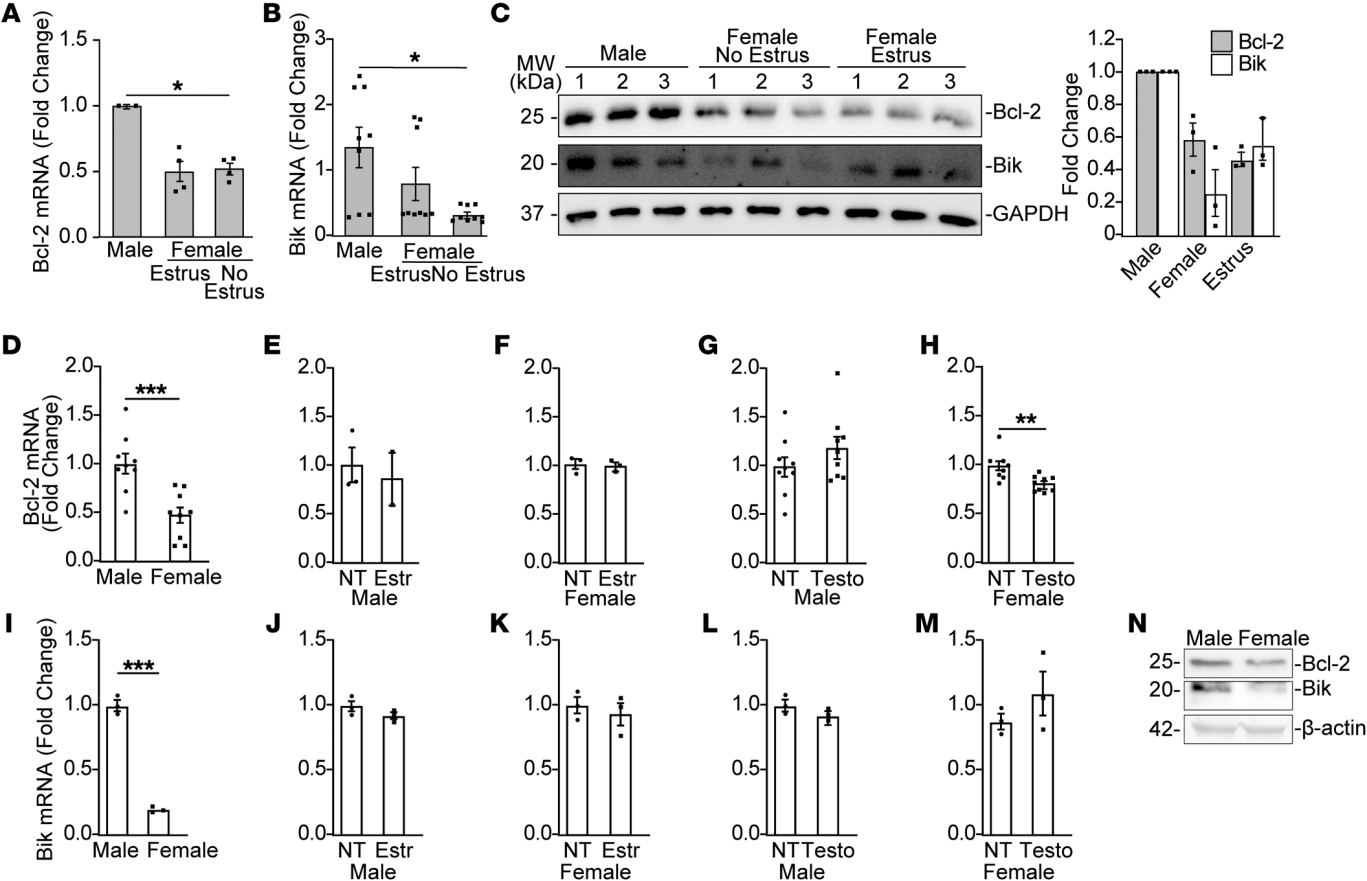
Bik and Bcl-2 levels are reduced in female airway cells. Lung tissues from male and female C57BL/6 mice were analyzed for (**A**) Bcl-2, (**B**) Bik mRNA, and (**C**) Bik and Bcl-2 protein levels by qRT-PCR and Western blotting. (**D**) Bcl-2 mRNA levels in male and female *bik^–/–^* MAECs. Bcl-2 mRNA levels in (**E**) male and (**F**) female *bik^–/–^* MAECs treated with vehicle or 10 nM estradiol and (**G**) in male or (**H**) female *bik^–/–^* MAECs treated with vehicle or 10 nM 5αDHT. Bik mRNA levels in male and female WT MAECs (**I**). Bik mRNA levels in (**J**) male and (**K**) female *bik^–/–^* MAECs treated with vehicle or 10 nM estradiol and in (**L**) male or (**M**) female *bik^–/–^* MAECs treated with vehicle or 10 nM 5αDHT. mRNA levels were analyzed by qRT-PCR. *n* = 3–9/group; *N* = 2. (**N**) Bcl-2 and Bik protein levels in male and female *bik^+/+^* MAECs analyzed from protein extracts by Western blotting. Two-tailed Student’s *t* test was used to compare between 2 groups, and grouped results were analyzed using 2-way ANOVA. Data are represented as mean ± SEM. *P < 0.05; **P < 0.01; ***P < 0.001.

Because lung inflammation under Bik-deficient conditions is more pronounced in the lungs of female mice than male mice, we compared Bcl-2 levels in the lungs of male and female mice. Bcl-2 and Bik mRNA levels (Figure 7, A and B) were reduced by approximately 2-fold in the lungs of female compared with male mice, irrespective of whether the female mice were in estrus or not. Similarly, Bcl-2 and Bik protein levels were reduced by approximately half in female compared with male mice (Figure 7C). To investigate whether Bcl-2 levels are reduced in female mice independently of Bik and whether hormones affect Bcl-2 expression, we compared Bcl-2 mRNA levels in male and female *bik^–/–^* MAECs and before and after treating with estradiol and 5α-dihydrotestosterone (5αDHT). Bcl-2 mRNA levels were reduced by 50% in nontreated *bik^–/–^* male compared with female MAECs (Figure 7D). Based on previous studies, we treated *bik^–/–^* male and female MAECs with 10 nM estradiol or 5αDHT and found that neither hormone greatly affected Bcl-2 mRNA levels (Figure 7, E–G), except for 5αDHT reducing Bcl-2 mRNA levels by approximately 20% in female *bik^–/–^* MAECs (Figure 7H). We also examined WT male and female MAECs and found that Bik mRNA levels were reduced by 80% in female compared with male MAECs (Figure 7I), but Bik mRNA was not affected by treatment with estradiol (Figure 7, J and K) or 5αDHT (Figure 7, L and M). Bcl-2 and Bik protein levels were also reduced in female compared with male MAECs (Figure 7N). These findings suggest that Bcl-2 and Bik expression are reduced in female cells independently of Bik and hormonal effects and that genetic effects are predominantly responsible for the reduction of Bik and Bcl-2 expression.

## Discussion

We identified a role for Bik in modifying the BH4 domain of nuclear Bcl-2 to interact with RPN1 and RPN2 and contribute to proteasomal degradation of p65 and its interacting proteins within the nuclear region of the cells. This report appears to provide the first evidence, to our knowledge, that regulating nuclear p65 levels can block not only low-grade inflammation, but also inflammation induced by CS, LPS, or allergens. We show that inflammation is enhanced in females compared with males because they express lower Bcl-2 levels. This investigation reveals a previously unappreciated aspect of the interplay between proteolysis and inflammation, with far-reaching implications for future research. We demonstrate that reducing inflammation in airway epithelia alone can rescue loss of the alveolar structures. These findings translate to humans who, due to a SNP in the intronic promoter region of the *BIK* gene, express reduced Bik levels, show higher inflammation in the circulating blood, and have a higher risk for decline in lung function during aging. Therefore, complete understanding of the molecular mechanisms that control nuclear p65 levels can provide early intervention strategies.

To our knowledge, this is the first report to demonstrate that Bik is localized to the nuclear region in epithelial cells in the absence of stimuli. Treatment with ABT-263 caused degradation of nuclear p65 in the absence of Bik, suggesting that ABT-263 can substitute for Bik in modifying the affinity of Bcl-2 to nuclear p65. Hence, modifying the BH3 groove of Bcl-2 using ABT-263 causes a conformational change in the BH4 domain that facilitates the interaction with p65. Mutations within the BH3 domain of Bik that render it nonapoptotic still did not affect the antiinflammatory nature of Bik, suggesting that suppressing inflammation is a nonapoptotic role for Bik.

As Bik levels are regulated by ubiquitination and proteasomal degradation (46), we postulate that the various roles of Bik, i.e., regulating ER Ca^2+^ release, inducing cell death by perforating mitochondria, or eliciting proteasomal degradation, are driven by careful regulation of Bik protein levels. We show that the main role of Bik, regardless of its phosphorylation status, is to localize Bcl-2 to the nucleus, modify the BH4 domain of Bcl-2, and thereby facilitate nuclear enrichment of the proteasome and affect stability of proteins, including that of p65.

Bcl-2 can be localized to the mitochondria or ER membranes (47, 48) or nucleus (49). Bcl-2 blocks the proapoptotic proteins Bax and Bak from forming pores on the mitochondrial outer membrane permeability (MOMP) (43). On the ER membrane, Bcl-2 interacts with IP_3_R to regulate the release of Ca^2+^ from ER stores (24, 50, 51). Bcl-2 has been reported to localize to the nucleus of neurons in aged F344 rats (52). Nuclear localization of Bcl-2 was also observed in cells from breast cancer, endometrial carcinoma, squamous cell carcinoma, and astrocytoma (53, 54). The nuclear localization of Bcl-2 depends on the phosphorylation status of T56, a process involving CDK1, PP1, and nucleolin (55). FK506-binding protein 38 (FKBP38), a mitochondrial chaperone, interacts with and targets Bcl-2 to the mitochondria (56), while Bcl-2 mutant lacking the BH4 domain fails to interact with FKBP38 and translocates to the nucleus (57). Nuclear Bcl-2 also sequesters glutathione (GSH) within the nucleus, likely impeding caspase activity triggered by granzyme B (58). However, the mechanisms governing Bcl-2 nuclear translocation as well as the role of nuclear Bcl-2 are poorly understood. In addition, Bik phosphorylation did not have any role in the binding to nuclear Bcl-2 and in Bcl-2 interacting with nuclear p65. The main role of nuclear Bik is to localize Bcl-2 to the nucleus, modify the BH4 domain of Bcl-2, and thereby facilitate nuclear enrichment of the proteasome and affect protein stability.

Previous studies showed that Bcl-2 WT and a mutant that lacked the BH4 domain (ΔBH4) localized to the nucleus in HEK293 cells and failed to protect cells from apoptosis, but inhibited transcription by AP1, CRE, and NFAT (59). Bcl-2–ΔBH4 increased nuclear NF-κB levels, leading the authors to assume that Bcl-2–mediated modification of IκBa could impede phosphorylation at the specific serine residues, modify ubiquitination, or enhance resistance to degradation (18). We also found that mutations within the BH4 domain enhanced Bcl-2/p65 interaction. Notably, we demonstrate that Bcl-2 regulates inflammation without affecting upstream degradation of IκBα, but rather by directly facilitating degradation of nuclear p65. If cytosolic p65 is released by a strong stimulus that causes degradation of IκBα, the large amount of p65 that enters the nucleus annuls the protective role of nuclear Bik/Bcl-2, as is seen when mice are given 50 μg LPS instead of 5 μg or when MAECs are treated with a strong inflammatory stimulus, such as TNF-α.

Mutations at the BH1 and BH4 domains or triple-phosphorylation mutants of Bcl-2 failed to degrade nuclear p65. Additionally, while the BH4 domain mutants failed to interact with nuclear p65, the BH1 mutant interacted with nuclear p65. This suggests that the BH4 domain is primarily involved in the degradation of nuclear p65. Because the triple-phosphorylation mutants failed to interact with and cause degradation of nuclear p65, phosphorylation of Bcl-2 at the 3 motifs appears to be required for Bcl-2–mediated degradation of nuclear p65. Ubiquitination of p65 occurs predominantly in the nucleus, and inhibition of proteasome activity selectively stabilizes nuclear p65 with minimal effect on the cytoplasmic fraction (60, 61). Bik does not affect nuclear translocation of p65, but only enhances p65 degradation in the nucleus, resulting in increased proinflammatory cytokine production. Regulation of inflammation solely by regulation of nuclear p65 levels that does not involve translocation of p65 may be an important mechanism for many chronic conditions.

**Figure 8:**
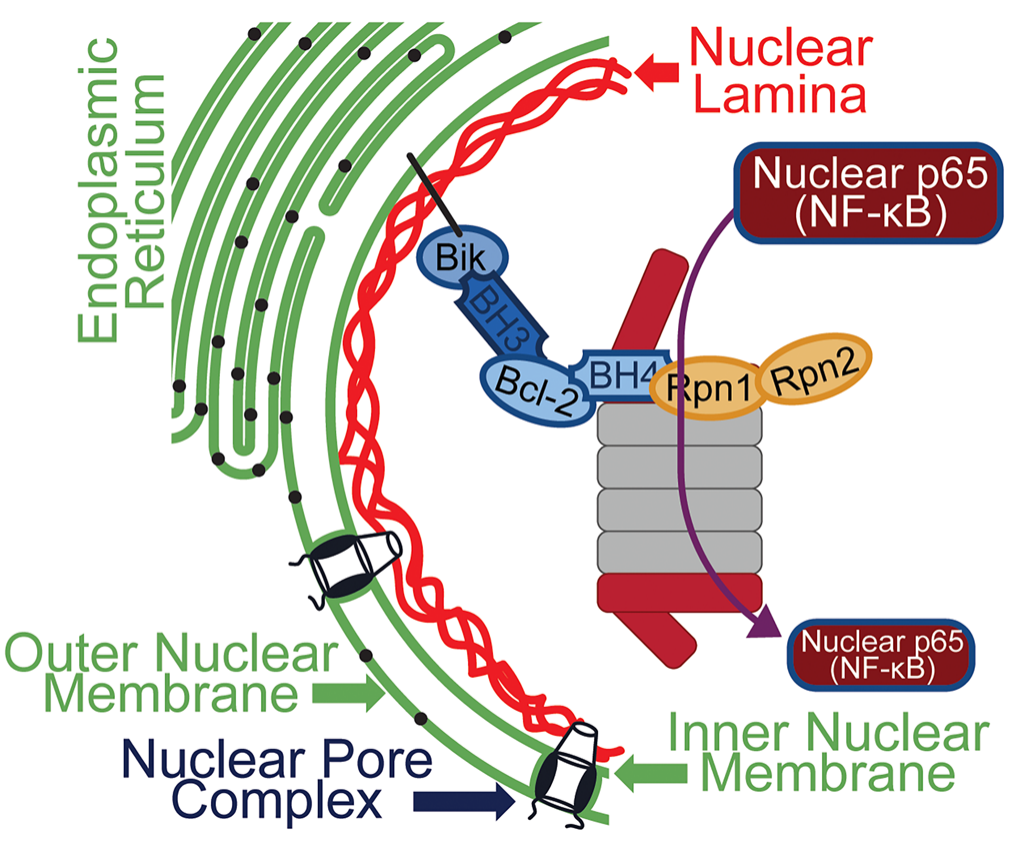
Schematic showing that Bcl-2 anchored to the nuclear membrane by interacting with Bik. Bik modifies the BH4 domain of Bcl-2 to interact with RPN1 and RPN2 and increases the activity of the nuclear proteasome to degrade nuclear p65.

Consistent with the proteomics data of immunoprecipitates from nuclear lysates of WT and Bik-deficient MAECs, the presence of Bik was required for Bcl-2 to interact with RPN1 and RPN2, confirming the role of RPN1 and RPN2 in Bik/Bcl2-mediated degradation of nuclear p65. The 26S proteasome is assembled from the 20S proteolytic core particle with 19S regulatory particles located at each end (62). The homologous subunits RPN1 and RPN2 are the largest subunits of the 19S particle and are crucial for both engaging with ubiquitin-binding proteins, such as RPN13 (63) and RAD23 (64), and acting as scaffolds for the preassembly of RPN1 together with the AAA-ATPase subunit (Rpt) heterohexamer precursor entities (65, 66). Our findings that the proteasomal inhibitor MG132 blocks Bik/Bcl-2–mediated reduction of nuclear p65 and inflammation together with the interaction Bik/Bcl-2/p65 with RPN1 and RPN2 supports the idea that Bik, acting through Bcl-2, may be crucial for nuclear localization of the proteasome (Figure 8).

Casein kinase 11 (CK11), which phosphorylates p65 at S536, promotes p65 degradation by an E3 ligase complex composed of Cullin2 and copper metabolism Murr1 domain 1 (COMMD1) to inhibit RIG-I/TLR signaling (67). In unstimulated cells, p65 Ser468 phosphorylation is regulated by glycogen synthase kinase 3 (GSK3) to negatively regulate basal NF-κB activity by facilitating the recruitment of an E3 ligase complex containing COMMD1, cullin2, and SOCS1 (68). Which E3 ligases are involved in the Bik/Bcl-2-facilitated degradation of p65 remains to be determined.

We found that subjects with the Bik GG genotype, i.e., reduced Bik levels, have increased inflammatory factors in the circulating blood. Further, female Bik-deficient mice showed increased lung inflammation when exposed to CS or LPS. We show that female mice express reduced Bcl-2 mRNA and protein levels, and this may explain why they are more susceptible to lung inflammation. The genotype effect is likely not sex dependent, as the genotype-dependent binding of IRF-1 reduces Bik levels regardless of sex hormones. However, the effect of Bik is sex dependent, as the Bcl-2 levels would be affected by sex. Our findings are supported by previous observations showing that the development of emphysema is more rapid in female compared with male mice exposed to CS chronically (69, 70). Similarly, female mice show increased serum levels of ovalbumin-specific IgG1 and IgE (71) and increased inflammation (72) when exposed to an allergen. In general, women experience increased morbidity and mortality from inflammatory lung diseases, such as asthma, COPD, and cystic fibrosis. Among smokers, women develop COPD after smoking fewer numbers of cigarettes per lifetime (i.e., fewer pack years of smoking) (73) and are 2 to 3 times more likely to experience hospitalization than are male patients (74). In patients with severe COPD and oxygen dependence, women have a 50% increase in the risk of mortality compared with men (75). These data suggest that female sex is a risk factor for morbidity and mortality in inflammatory lung diseases. As a result, several studies have explored the possibility that sex-related hormones are involved in mediating disease progression in asthma and COPD. Airway inflammation related to allergens was diminished in ovariectomized rats and restored to levels found in intact females with estrogen replacement in the ovariectomized rats (76), suggesting the role of female hormones in augmenting allergen-induced airway inflammation. Interestingly, reduction of Bcl-2 and Bik levels in females is not due to hormonal effects, but is due to genetic effects, as nontreated female compared with male MAECs expressed reduced levels of Bcl-2 and Bik mRNA and protein. The molecular mechanisms underlying this sex-specific difference needs further investigation.

The inducible expression of Bik in mice was restricted to the airway epithelium by the CCSP promoter, and these mice were bred into the *bik^–/–^* background. Therefore, the inhibition of HDM- or LPS-induced inflammation was driven by Bik expression in only the AECs. Restoring Bik expression in the airways prevented the development of emphysema. Loss of alveolar epithelial cells is observed in Bik-deficient settings, where the apoptotic Bik is absent. If the apoptotic role of Bik was needed to kill alveolar cells, emphysema should occur in the WT mice. Therefore, it is the inflammation present in Bik-deficient mice that is causing destruction of the alveolar structures. The initiating role of the airway epithelium to induce lung inflammation has been proposed (77) and is supported by findings in human studies (78). However, our studies support the hypothesis that the initial event may involve inflammation in the small airway epithelium, and such experimental evidence showing causation has not been reported.

Antiinflammatory factors that keep low-level inflammation in check at young ages may be absent or weakened during the aging process. We show that reduced Bik expression in humans was caused by a genetic variant in the intronic region of the *BIK* gene that suppresses Bik levels by 2-fold due to IRF-1 binding. Both people with the *Bi*k GG compared with the *Bik* AA genotype and *Bik*-deficient mice showed increased inflammatory cytokines, suggesting that reduced Bik levels result in accelerated emphysema development and decline in lung function. Previous reports in human COPD have reported that inflammation that emanates from small airways may be responsible for the decline in lung function and ultimate destruction of the alveolar structures (28, 79). Increasing Bik to appropriate levels in at-risk patients with the GG genotype may be an attractive therapeutic strategy for preventing accelerated lung function decline and COPD.

## Methods

### Study cohorts.

The protocols for the recruitment of participants and the collection of data in the LSC, FHS Offspring cohort, ECLIPSE cohort, and COPDGene Study have been described previously (32–36). All of the studies were approved by an institutional review board or ethics committee, and participants provided written informed consent.

### Human plasma samples.

Specimens and data obtained from the Lung Tissue Research Consortium (LTRC) were used in a manner that protects the privacy and confidentiality of the tissue donor subjects. Genotyping of 400 DNA samples was initially performed by allelic discrimination as previously described (80).

### Primary HAECs.

Primary HAECs were obtained by bronchoscopy from cancer-free current or former smokers or from discarded lung specimens.

### Study approval.

All animal studies were approved by the institutional animal care and use committees at the Lovelace Biomedical Research Institute and at Brigham Women’s Hospital and were performed at both the Lovelace Respiratory Research Institute, Albuquerque, New Mexico, USA, and Brigham and Women’s Hospital, Boston, Massachusetts, USA, facilities approved by the Association for the Assessment and Accreditation for Laboratory Animal Care International. All human studies and the use of primary HAECs were approved by the institutional review board or ethics committee at the Lovelace Biomedical Research Institute and by the Institutional Review Board of Mass General Brigham, Somerville, Massachusetts, USA (IRB protocol 2020P000254). Human lungs unsuitable for transplantation were obtained under a protocol approved by the University of North Carolina at Chapel Hill Office of Human Research Ethics. All participants provided written, informed consent.

### Data availability.

Values for all data points in graphs are reported in the Supporting Data Values file. The ECLIPSE data set analyzed in the current publication is based on the use of study data downloaded from the NCBI’s Database of Genotypes and Phenotypes (dbGaP phs001252.v1.p1.c1).

Further information is available in Supplemental Methods.

## Author contributions

YT designed research studies. YAM, JTJ, ZHN, MR, and JRQ conducted experiments. CL, GTO, WG, JD, MC, and AAL analyzed data. SR provided reagents. YAM, JTJ, and YT wrote the manuscript. CL, ZHN, MR, JRQ, GTO, WG, JD, MC, AAL, and SR revised the manuscript.

## Supplementary Material

Supplemental data

Supporting data values

## Figures and Tables

**Table 2:**
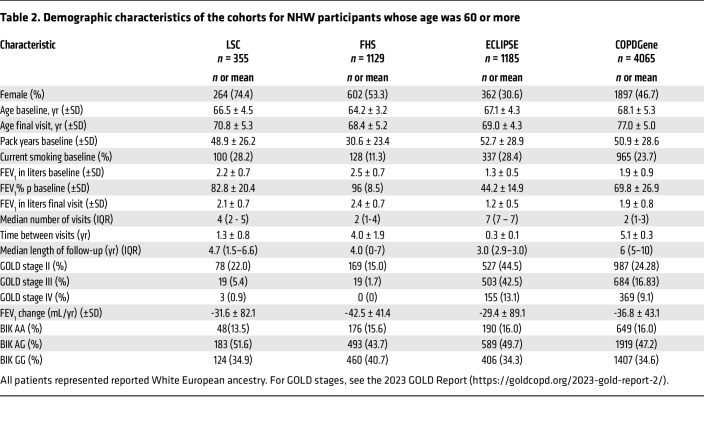
Demographic characteristics of the cohorts for NHW participants whose age was 60 or more

**Table 1:**
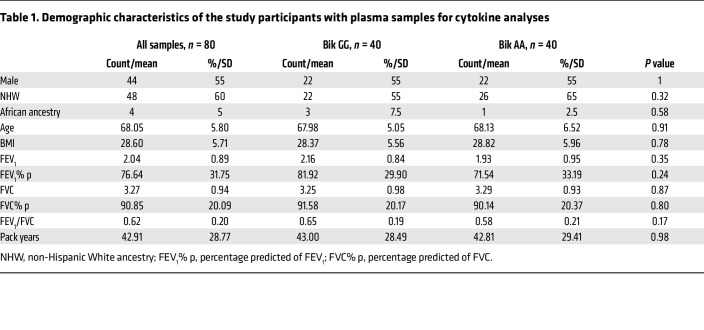
Demographic characteristics of the study participants with plasma samples for cytokine analyses

## Version 1 12/19/2023

In-Press Preview

## Version 2 02/15/2024

Electronic publication
