# Supplementary material for: Bik promotes proteasomal degradation to control low-grade inflammation
Source: J Clin Invest. 2023 Dec 19;134(4):e170594. doi: 10.1172/JCI170594 (PMC10866658; doi:10.1172/JCI170594)
Supplement: Supplemental data [file jci-134-170594-s223.pdf]

Supplemental Information

Bik promotes proteasomal degradation to control low grade inflammation

[1*]Yohannes A. Mebratu, [2*]Jane T. Jones, [1]Congjian Liu, [1]Zerihun H. Negasi, [1]Mizanur Rahman, [1]Joselyn Rojas Quintero, [4#]George T. O'Connor, [5]Wei Gao, [5,6]Josée Dupuis, [7]Michael Cho, [8]Augusto A. Litonjua, [9]Scott Randell, [1]Yohannes Tesfaigzi

Methods

Study Cohorts

All participants included in these analyses were of non-Hispanic white ancestry or White European Heritage race category and age greater and equal to 60 years old. Spirometry was performed in accordance with American Thoracic Society recommendations.(78) All of the studies were approved by an institutional review board or ethics committee, and participants provided written informed consent.

Human plasma samples: Specimens and data obtained from the Lung Tissue Research Consortium (LTRC) were used in a manner that protects the privacy and confidentiality of the tissue donor subjects. Genotyping of 400 DNA samples was initially performed by allelic discrimination as previously described (79).

Primary human airway epithelial cells: HAECs were maintained in complete SAGM media (Lonza) containing TGFβ antagonist, BMP4 antagonist, WNT agonist, and ROCK inhibitor (Walkersville, MD) in a 37°C incubator supplied with 5% CO_2 for 1 or 2 passages. Other sets of HAECs were differentiated at air liquid interface on Transwell membranes (Corning) as previously described (80).

Animals: C57Bl/6 mice were introduced into animal protocol at 7-8 weeks old. The $bik^{+/-}$ mice on C57BL/6 background were provided by Andreas Strasser (Walter and Eliza Hall Institute, Melbourne, Australia). $Bik^{+/+}$ with $bik^{-/-}$ littermates were bred from the respective heterozygote mice at the Lovelace Respiratory Research Institute under specific pathogen-free conditions and genotyped as described (20). The conditional and airway specific overexpression of Bik was achieved by mating two lines of transgenic mice, the CCSP–reverse tetracycline responsive transactivator (CCSP-rtTA) mice, bearing the rtTA under the control of the CCSP gene promoter and the tetracycline operator $(TetO)_7$–Bik mice, containing TetO and minimal cytomegalovirus (CMV) promoter and the Bik transgene. $(TetO)_7$-Bik mice were generated at the M.D. Anderson

Genetically Engineered Mouse Facility following standard methods and Institutional Animal Care and Use Committee-approved protocols and bred with CCSP-rtTA mice at LRRI. To conditionally express Bik in the lung, transgenic mice were fed with doxycycline-containing diet.

Exposures of Mice to Cigarette Smoke or Lipopolysaccharide (LPS): *Bik*$^{+/+}$ and *bik*$^{-/-}$ littermates were bred from the respective heterozygote mice at the Lovelace Respiratory Research Institute under specific pathogen-free conditions and genotyped as described previously (20). Male and female *bik*$^{+/+}$ and *bik*$^{-/-}$ mice at 6 - 8 wk of age were exposed to CS for 6 h/d, 5 d/wk, for 3 weeks, in H1000 chambers as described (64). For all experiments, type 2R4F research cigarettes (Kentucky Tobacco Research and Development Center) were used. Exposure concentrations were kept at 250 mg TPM/m^3 except for the first wk of exposure, during which the mice were exposed to 100 mg TPM /m^3. Control animals were housed in similar exposure chambers and exposed to filtered air (FA).

For LPS (LPS, Sigma) exposures, mice were lightly anesthetized with isoflurane and intranasally instilled with 5 or 50µg LPS. Mice were euthanized four hours following LPS instillation. For the house dust mite (HDM, Greer) exposures, TetoBik- and tetoBik+ mice were instilled intranasally with 50 µg *Dermatophagoides Pteronyssinus* and BAL fluid was analyzed for neutrophil numbers 5 days later. *bik*$^{-/-}$ mice were instilled with 50 ug HDM intranasally daily for 5 consecutive days. On days 6 and 7, mice were intranasally treated with 10 µM of control TAT peptide, BH3 WT Bik peptide, or BH3 mutant Bik peptide. BAL fluids were analyzed for inflammatory cell numbers. In a separate experiment, mice were sensitized with HDM via intranasal instillation on days 1 and 8 and subsequently challenged with HDM 5 d/wk for 4 wks and kept on doxycycline diet during this time (32).

Six weeks old (TetO)$_7$–Bik mice and their littermates were kept with 400 mg/l doxycycline water until they reach the age of 25 weeks. Lung tissues were harvested, inflated, and fixed with zinc

formalin. H&E-stained lung tissues were analyzed for changes in alveolar space using ImageJ software.

Tissue Processing, Lavage and Lung Morphology: Mice were euthanized, tracheas cannulated, left main stem bronchi temporarily clamped, and right lungs lavaged three times with 0.5 ml phosphate-buffered saline (PBS). The bronchoalveolar lavage fluid (BALF) was assessed for the number of inflammatory cells and the non-cellular supernatant cytokines for levels of chemokines and cytokines. The left lung was inflated with 4% buffered formalin at a constant hydrostatic pressure of 25 cm for 6 h. Lungs were fixed further by immersion in fixative for 48–96 h. The fixed left lung lobes were trimmed, processed for histology, sectioned, and stained with hematoxylin and eosin (H&E) as described.(81) Briefly, depending on the size of the lung, four or five slices were prepared and numbered from the proximal (slice 1) to the distal (slice 4 or 5) end. The slices were then embedded in paraffin and tissues sections (5 μm thick) prepared and stained with Alcian Blue and Hematoxylin-Eosin (Sigma, St. Louis, MO, USA) as per manufacturer's instructions. Emphysema was described as irregular, multifocal expansion of alveolar airspaces and alveolar ducts due to destruction of parenchymal tissue. Morphometry was used to quantify emphysema as described previously.(82) Digital images of the H&E stained slides were captured using an Olympus NanoZoomer (Hamamatsu Photonics) slide scanner with a 20x objective lens to scan the slides. Volume-weighted mean volumes of alveoli were estimated by the method of point sampled intercepts (83). This method combines measurements of both mean volume and variability of size of the specified parenchymal spaces. The volume weighted mean volumes of alveoli can be estimated on a single section and the estimation is unbiased without shape assumptions (84, 85). The VisioMorph module of VisioPharm analysis software (VisioPharm, Denmark) was used to determine alveolar volume or mean cord length.

BAL Cell Counts: BAL was centrifuged at 1000g at 4°C for 5 minutes and supernatant was collected and stored in aliquots at -80°C for cytokine analyses. The cell pellet was diluted in 5 ml

of media and BAL cell number was enumerated using a hematocytometer, cytospin slides were prepared with 50,000 cells, stained with hematoxylin/Giemsa, and cell differential quantified as described previously (86).

RNA Isolation and qRT-PCR: RNA was isolated from HAECs using TRI reagent (Sigma-Aldrich, St. Louis, MO) and mRNA was isolated following manufacturer's instructions. cDNA was synthesized using the high-capacity cDNA reverse transcription kit (Applied Biosystems, Foster City, CA). TaqMan real time PCR was conducted to measure the expression of candidate genes using the ΔCT method with CDKN1β, β-Actin, or 18s as the endogenous control. The expression data are presented as the average from 3 cDNAs independently made from one mRNA sample.

Cell culture: A549 cells (purchased from American Type Culture Collection, Manassas, Virginia) stably transfected with the NF-κB luciferase reporter construct (Panomics) were cultured in DMEM media containing 10%FBS, L-glutamine, penicillin/streptavidin, and selected in hygromycin (100μg.ml). HEK293T cells were also cultured in DMEM media containing 10%FBS, L-glutamine, and penicillin/streptavidin. Primary mouse airway epithelial cells (MAECs) were isolated from murine trachea as previously described (87). Briefly, tracheas were removed from the animal post-mortem and incubated overnight 4°C in a solution containing DNAse (0.1mg/ml) and Pronase (1.4 mg/ml). The next day, MAECs were washed with HAMs F12 media (Gibco), MAECs and primary human airway epithelial cells (HAECs) were plated in small airway epithelial media supplemented with 1μM A8301 (TGFβ antagonist), 1μM DMH-1 (BMP4 antagonist), 1μM CHIR99021 (WNT agonist), and 10μM Y27632 (ROCK inhibitor), all purchased from TOCRIS as previously described (88).

Mouse epithelial airway cells (MTEC) from male and female 12 week old *bik*$^{+/+}$ and *bik*$^{-/-}$ mice grown in Ham's F-12 media (Gibco, cat- 11765054) supplemented with SAGM airway epithelial growth bulletkit (Lonza, Cat-CC3118) in 12-well plates. At 70-80% confluency, cells were exposed to 5,10,20, 50 or 100 nM of 5alpha-dihydrotestosterone (testosterone) (Millipore Sigma, Cat-01-

001-193) or β-estradiol (Sigma Aldrich, cat-E2758) for 24 h. Cells were washed with PBS and total RNA was extracted by Qiagen mini RNA extraction kit (Qiagen, cat-74104) and of Bcl-2 or Bik mRNA levels were determined by qRT-PCR.

Immunoblotting and Immunoprecipitation: Cytosolic and nuclear extracts were isolated as previously described (20). Briefly, cells were lysed in the presence of a buffer containing 10mM Tris (pH 8), 60mM KCL, 1mM EDTA, 0.5%NP-40, 1mM DTT, and a protease inhibitor cocktail (Sigma). Following centrifugation at 2000g, supernatants were collected (cytosolic) and pellets were subjected to high salt buffer extraction (20mM Tris pH 8, 420 mM NaCl, 0.2 mM EDTA, 1.5 mM $MgCl_2$, 25% glycerol, 1mM DTT, and a protease inhibitor cocktail). After centrifugation at 10,000g, supernatants were collected as the nuclear fraction. To isolate cytosolic, mitochondrial, ER, nuclear, NP-40, and nuclear pellet, a fractionation protocol was utilized. Briefly, cells were trypsinized and collected by centrifugation. They were then resuspended in a hypotonic buffer (10 mM HEPES, 1mM EGTA, and 25mM KCL) for 10 minutes and centrifuged (supernatant was removed and discarded). Cells were then resuspended in an isotonic buffer (10mM HEPES, 250 mM Sucrose, 1mM EGTA, and 25 mM KCL) and Dounce homogenized. After centrifugation, the pellet (nuclear) was incubated in a hypertonic buffer (50 mM HEPES, 50mM KCL, and 300 mM NaCl) an NP-40-based buffer (20 mM Tris pH 8, 100 mM NaCl, 1% NP-40, 3 mM EDTA, 2 mM DTT, and a protease inhibitor cocktail), and the final pellet was dissolved in a buffer containing SDS (2%) and DTT (50 mM). Meanwhile, the supernatant after the Dounce homogenizer step was centrifuged at 28,000 rpm for 10 minutes and the pellet saved (mitochondrial pellet). The supernatant was then centrifuged at 48,000 rpm for 1 hour. The supernatant was designated as cytosolic fraction and final pellet was designated as ER fraction.

To isolate soluble nuclear fractions, we sequentially lysed cell components by extracting the cell fractions with buffers involving various detergents and ionic strength. Briefly, cell pellets were first incubated on end-over-end rotator for 10 min at 4 °C with ice-cold lysis buffer A (1 mM MgCl2,

1mM Na3VO2, 10 mM KCl, 50 mM HEPES (pH 7.4), 1 M Hexylene glycol, 1% protease inhibitor cocktail). Following centrifugation at 2000g for 10 min, supernatants were collected (cytosolic). The nuclear pellet #1 was incubated on ice with ice-cold lysis buffer (10 mM NaCl, 1 mM MgCl2, 10 mM KCl, 50 mM HEPES (pH 7.4), 1% Igepal, 1 M Hexylene glycol, and protease inhibitor cocktail) for 30 min and centrifuge at 7000 *g* for 10 min at 4 °C to collect the nuclei. The supernatant is collected as perinuclear fraction #1. This fraction contains the proteins that are loosely associated with the nuclei. The nuclear pellet #2 was resuspended in lysis buffer containing 150 mM NaCl, 50 mM HEPES, 1% Igepal, 1 M Hexylene glycol, and protease inhibitor cocktail, incubated on ice for 30 min, and centrifuge at 7000 × *g* for 10 min at 4 °C to collect the nuclei. The supernatant was collected (perinuclear fraction# 2). This fraction contains the proteins that are tightly associated with the nuclei. Finally, the nuclear pellet #3 was incubated on ice for 30 min with buffer containing 400 mM NaCl, 50 mM HEPES, 0.5% sodium deoxycholate, 0.15 sodium dodecyl sulfate, 7 µL of benzonase and protease inhibitor cocktail and resuspend. The sample was centrifuged at 7800x*g* for 10 min at 4 °C to remove the nuclear proteins from the non-soluble pellet. The supernatant was collected, and this fraction contains the nuclear proteins and was used to analyze proteins by Western blot.

Protein samples were immunoprecipated using protein A agarose beads (Catalogue no. 9863P, Cell Signaling Technology) conjugated to p65 or Bcl-2 antibody according to manufacturer's instructions (Thermo Scientific). All samples were separated by SDS-PAGE and subsequently analyzed by the following antibodies: Bik (Catalogue no. ab52182, Abcam, Rabbit polyclonal; 1:1000), and Lamin (# ab238303; mouse monoclonal; clone 4C11; 1:1000; Abcam); p65 (#8242; Rabbit mAb, clone D14E12; 1:1000; Cell Signaling Technology); p50 (Abcam; ab32360,Rabbit mAB; Clone E381); 1:2000); IκBα (#ab32518; Rabbit mAb; clone E130; 1:1000; Abcam); phospho-IκBα (mouse mAb, #9246s; clone 5A5; Cell Signaling Technology); Bcl-2 (Mouse mAb, #ab238303; clone 4C11, 1:1000, Abcam); β-Actin (Mouse mAb, Catalogue n. 3700; clone 4170;

1:5000, cell Signaling Technology); IRF-1 (Mouse mAb; #sc-74530; clone H-8; Santa Cruz); RPN1 (PSMD2) (Rabbit polyclonal; #PA5-27663 1:,2000; ThermoFisher); RPN2 (PSMD1) (Rabbit polyclonal, ab2941; 1:5000; Abcam); HA (Mouse mAb; # 2367; clone 6E2, Cell Signaling Technology); Flag (Rabbit mAb, #14793; clone D6W5B; 1:1000; Cell Signaling Technologies) GAPDH (Rabbit mAb # 5174; clone D16H11, Cell Signaling Technology); β-catenin (Rabbit mAb #9582; clone 6B3; Cell Signaling Technology); Lamin B1 (Rabbit mAB #13435; clone D9V6H; Cell Signaling Technologies)

Reagents: The following peptides were purchased from Anaspec with the following sequences: TAT: H-GRK KRR QRR RPQ; TAT-Bik: H-GRK KRR QRR RPQ ALA LRL ACI GDE MD-OH; TAT-mutBik: H-GRK KRR QRR RPQ ALA LRG ACI GDE MD-OH. Cells were treated with peptides at the indicated doses for the indicated times. Cells were also treated with the small molecule inhibitor, ABT-263 (AbbVie, Inc.), for the indicated times and doses. The following plasmids utilized were a gift from Dr. David Andrews: Empty Vector, WT Bcl-2, and ER-targeted Bcl-2. ER-targeted Bcl-2 construct was generated by exchanging the Bcl-2 carboxyterminal membrane-insertion sequence for an equivalent sequence from cytochrome b5, a DNA encoding the analogous 35 amino acid sequence of the ER-specific isoform of rat hepatic cytochrome b5 (89). Transfections were performed with Trans-IT 2020 (Mirus) according to manufacturer's instructions.

Immunofluorescence: A549 cells, MAECs, or HAECs were fixed in 4% paraformaldehyde for 20 minutes, permeabilized in 1% triton, blocked in 1% BSA, and incubated in the following primary antibodies: Bik (Abcam; ab52182; Rabbit polyclonal; 1:1000), p65 (mouse mAb, # sc-8008; clone F-6; 1:500; Santa Cruz), and RPN1 (Rabbit polyclonal; #PA5-100323; 1:200; ThermoFisher); RPN2 (PSMD1) (ab2941, Rabbit polyclonal, 1:500; Abcam). Cells were subsequently stained by anti-rabbit Alexa 647 (Polyclonal; #A-21244; 1:200; Invitrogen), or anti-rabbit Alexa 594 (Polyclonal; # A11012; 1:1000; ThermoFisher), mounted with glass coverslips using

Fluoromount-G (Southern Biotech) and immunofluorescence was detected using Axioplan 2 (Carl Zeiss) with a Plan-Aprochromal 63x/1.4 oil objective and a charge-coupled device camera (SensiCam; PCO) using an acquisition and processing software Slidebook 5.5 (Intelligent Imaging Innovation, CO).

Promoter Activity Assays: Genomic PCR fragment from the promoter region of *BIK* (1947 bp) was cloned into *Kpn*I and *Nhe*I sites of the pGL3-basic luciferase reporter vector (Promega Corp., Madison, WI, USA), to make the pPRO construct. Next, a 750bp conserved region containing rs738276 was cloned immediately downstream of the promoter using the *Nhe*I and *Bgl*II sites, with either rs738276 A or G allele (p750A and p750G). The endogenous *BIK* intron 1 splice acceptor was next cloned into the p750A/G constructs to give the p750A/G-SA constructs. Constructs containing the rs738276 G allele were developed by initial PCR amplification from an individual with the rs738276 A allele (A:T base-pairing), then site-directed mutagenesis of this base position to a G:C base pairing in subsequent clones, using a PCR based method(90). All constructs were confirmed by Sanger sequencing, and sequences of all primers used for cloning are contained in **Supplementary Table 1**.

The dual luciferase reporter (DLR) assay (Promega, Madison, Wisc.) was used to test the impact of rs738276 alleles on promoter activity. Plasmids were co-transfected with the *Renilla luciferase* plasmid for normalization purposes. Cells were grown in 24-well culture plates until 70-80% confluent. The plasmids were transfected with TransIT-2020 (Mirus Bio, Madison, Wis.) using manufacturer’s protocol, and harvested and assayed for luciferase activity twenty-four hours after transfection.

The *BIK* gene comprises four introns and five exons spanning a region of about 20 kb, with a total unprocessed transcript length of 18.97kb (including UTRs). The noncoding first exon precedes a 13.2kb first intron that comprises the majority of the unprocessed transcript. A promoter region for *BIK* was defined based on ChIP-seq data from the ENCODE project that indicates a 1947 bp

region roughly symmetrical about the transcription start site with a H3K4 tri-methylation, DNase hypersensitivity, and binding of multiple transcription factors in various cell types (**Supplementary Figure 3A**), consistent with the presence of a promoter. Similar to the promoter region, the 750bp intronic region where rs738276 is localized, demonstrates a conservation across all placental mammals (**Supplementary Figure 3A**) that is comparable to the exonic regions (not shown). Because rs738276 was not in LD with other SNPs and was located within a potential promoter region, we cloned the region upstream of a luciferase reporter gene to investigate its function.

Three luicferase reporter constructs were tested: (1) The ~2kb upstream promoter region and terminating at 980 from the transcriptional start site (pPRO). (2) The 750bp conserved sequence immediately downstream of the pPRO promoter with either rs738276 A or G allele (p750A/G). (3) The endogenous *BIK* splice acceptor at the end of the 750bp sequence of p750A/G resulting in splicing of intronic sequence (p750A/G-SA) (**Supplementary Figure 3B**). The point mutation A/G was constructed by site-directed mutagenesis and verified by sequencing. The pPRO construct demonstrated strong promoter activity relative to the empty vector in a variety of cell types of lung epithelial origin; ranging from 28-fold increase in N1 cells to 493-fold in H1299 (**Supplementary Figure 3C**). Compared with previously identified *BIK* promoters that comprised the 2 kb upstream region and ending at basepair +203 or -78 relative to the transcription start site (91) the pPRO construct showed a drastically increased promoter activity (**Supplementary Figure 3C**). These findings point to the importance for transcriptional activity of the *BIK* intronic region that is located between +203 to +980 from the transcriptional start site. However, luciferase activity was drastically reduced when the p750A/G constructs were inserted downstream of the pPRO sequence, likely due to the introduction of an inappropriate translation start site (data not shown). However, inserting the endogenous intron 1 *BIK* splice acceptor downstream of the 750bp conserved region (p750A/G-SA) restored the luciferase activity to that observed in pPRO construct (data not shown).

Proteomics Analysis: Mouse airway epithelial cells from *bik*$^{+/+}$ and *bik*$^{-/-}$ mice were grown to 100% confluency. Nuclear fractions were isolated after removing the cytosolic and perinuclear fractions and immunoprecipitated using anti-Bcl-2 antibody. Immunoprecipitates were run on SDS-PAGE gels and protein bands were cut and submitted for proteomic analysis. Identified peptides were selected based on their highest representation. Volcano plot was performed to identify the top proteins

Electrophoretic Mobility Shift Assay (EMSA): EMSAs were performed using the LightShift Chemiluminescent EMSA Kit (Thermo Scientific) as described by the manufacturer. Briefly, nuclear extracts from H1299 and AALEB cells were prepared, and 10 µg of each extract was incubated at room temperature in 10 mM Tris-HCl, 50 mM KCl, 2.5% (vol/vol) glycerol, 5 mM $MgCl_2$, 50 ng/µL poly(dI-dC), and 0.05% (vol/vol) Nonidet P-40 binding buffer, pH 7.5. After 10 min incubation at room temperature, 20 fmol of biotin-labeled oligonucleotide probes with the A allele (5'-/Biosg/CTA GGG CCA AAC TGA AAA GCT-3' and 5'-AGC TTT TCA GTT TGG CCC TAG-3') (Oligo-A) or G allele (5'-/Biosg/CTA GGG CCA GAC TGA AAA GCT-3' and 5'-AGC TTT TCA GTC TGG CCC TAG-3') (Oligo-G), or to the IRF-1 gene (5'-/Biosg/CTA GGG GGA AAC TGA AAC CCT-3' and 5'-AGG GTT TCA GTT TCG CCC TAG-3') (OligoIRF-1) was added to the equilibrated protein. For competition experiments, 10-1000-fold excess of unlabeled double-stranded probes was added prior to the labeled probe. Supershift assays were performed by pre-incubating the reaction mixture with 2 µg of IRF-1 antibodies (catalogue no. sc-49, Santa Cruz Biotechnology). The samples were resolved on a 6% non-denaturing polyacrylamide gel prepared in 45 mM Tris-borate and 1 mM EDTA (TBE) buffer. The specimens were electrotransferred onto a 0.45-µm Biodyne B nylon membrane (Pall Corporation) at 380 mA for 30 min at 4°C, and crosslinked to the membrane using a TL-2000 UV Translinker (Ultra-Violet Products). The blots were developed using Chemiluminescent Nucleic Acid Detection Module Kit (Thermo Scientific).

Chromosome Immunoprecipitation Assay: Primary HAECs from individuals homozygous for either rs738276 A or G allele were grown on 10 cm culture plate. At 75% confluency, cells were fixed with 1% formaldehyde, and the reaction was quenched using 1.25M glycine and scraped into cold PBS containing protease inhibitors. ChIP was performed using the Magna ChIP G kit (MAGNA0002; EMD Millipore) as described by the manufacturer. Briefly, immunoprecipitation was performed by incubating sonicated nuclear fractions overnight at 4°C with 10 µg of an anti-IRF-1 (catalogue no. sc-497) and normal mouse immunoglobulin G (IgG; sc-2025) (both from Santa Cruz Biotechnology). The genomic DNA fragments in the immunoprecipitated samples were analyzed by PCR by using a primer sets for the *Bik* intronic region (Forward primer 5′-TACAAAACACCACTGGGCCT -3′ and reverse primer 5′- GAGGGGGCGGTCAAGAATAC -3′), and 10 kb upstream of the *BIK* gene (Forward primer 5′-GTGTTGGGGCTGATAGACCA -3′ and reverse primer 5′- TCAGGGCACTCTGGGAAAGA -3′ as negative control. The primers used for the ChIP assays are listed in **Supplementary Table 2**.

Luminex Cytokine Assay: The cytokines in cell culture media were quantified by Luminex instrument (Luminex Corp.) using Multiplex Fluorescent Bead-Based Luminex Cytokine Assays (EMD Millipore).

Transmission Electron Microscopy: To visualize perinuclear region of cells with higher magnification, mouse lungs were fixed overnight in a mixture of 1.25% formaldehyde, 2.5 % glutaraldehyde, and 0.03% picric acid in 0.1 M sodium cacodylate buffer, pH 7.4. The fixed tissues were washed with 0.1 M sodium cacodylate buffer and post-fixed with 1% osmium tetroxide/1.5% potassium ferrocyanide for 2 h. Samples were washed in a maleate buffer and post fixed in 1% uranyl acetate in maleate buffer for 1 h and rinsed in ddH_20 and dehydrated through a series of ethanol (50%, 70%, 95%, (2x)100%) in water for 15 minutes in each solution. Dehydrated tissues were placed in propylene oxide for 5 min before they were infiltrated in epon mixed 1:1 with propylene oxide overnight at 4°C. The epon resin was polymerized in a 60°C oven

for 48 h. Tissues were then sectioned into 80 nm thin sections and imaged using a 1200EX Transmission Electron Microscope (JEOL, Peabody, MA).

Statistics: Data from at least 6 mice per group were presented as the mean ± standard error from the mean (SEM). Statistical analyses were performed using GraphPad Prism Software 5.0 (GraphPad Software, Inc., San Diego, CA). Two-tailed student t-test was used to compare between 2 groups. For all experiments, grouped results were analyzed using one-way or two-way analysis of variance (ANOVA). When significant main effects were detected ($p < 0.05$), a post test of multiple comparisons was performed (Tukey) to determine differences between treatment groups.

The association between *BIK* genotype (rs738276) and longitudinal measure of FEV1 in LSC, FHS, ECLIPSE, and COPDGene Study was assessed using linear mixed effects model with random intercept with adjustment for age, sex, pack-years, smoking status, and height at baseline visit, time since enrollment at each PFT test and principal components (6): The model included: Fev1 ~ sex + Pack years +Smoking_status+Height +Age +Follow-up years + Recessive/additive model + Follow-up years * recessive/additive genotypel + PCs, where FEV_1, Pack years, Smoking_status, and Height are time-dependent. The additive model is comparing between GG / GA / AA of BIK and using GG as reference group.

We combined results using a random-effects meta-analysis, evaluating the effect of *BIK* genotype on lung function decline by years of follow up. All statistical tests and plots for LSC, ECLIPSE and COPDGene studies were performed in R (version 3.6.3). The analyses for the FHS were performed in R (version 3.5.1) using the pedigreemm package (version 0.3.3) to account for familial correlation.

Study Approval: All animal studies were approved by the Institutional Animal Care and Use Committee and were performed at both the Lovelace Respiratory Research Institute, Albuquerque, NM and Brigham and Women's Hospital, Boston, MA facilities approved by the

Association for the Assessment and Accreditation for Laboratory Animal Care International. All human studies and the use of the primary HAECs were approved by the respective institutional review boards or ethics committee and by the Institutional Review Board of Mass General Brigham (IRB Protocol # 2020P000254). All participants have provided a written informed consent.

Supplementary Table 1: Primers used for cloning

Oligo name	Sequence	Used for
Promoter forward	5′-ATATAGGTACCCCCTCCCCTAATCTCTGAGG-3′	pPRO
Promoter reverse	5′-ATATAGCTAGCGGCTCCCAGACCACAGTAAA-3′	pPRO
750bp conserved forward	5′-ATATAGCTAGCGGGCTAACGTGAGACTCCAT-3′	p750 A/G
750bp conserved reverse	5′-ATATAAGATCTGACAGGGACCCTCACTCAAA-3′	p750 A/G
Splice acceptor forward	/5Phos/GATCTCCCAGACTGCTCAGTTCTTAGGGGTCCAGTCATATGCTGTCTTTTTGCCCCAGAGGAGAAAC	p750-SA A/G
Splice acceptor reverse	/5Phos/CATGGTTTCTCCTCTGGGGCAAAAAGACAGCATATGACTGGACCCCTAAGAACTGAGCAGTCTGGGA	p750-SA A/G
rs738276 SDMutageneis-F	5′-CCCAGTTAGCCAGACTGAAAAGCCA-3′	site directed mutagen. of rs738276
rs738276 SDMutagenesis-R	5′-TGGCTTTTCAGTCTGGCTAACTGGG-3′	site directed mutagen. of rs738276

Supplementary Table 2: Primers for the EMSA and ChIP Assays

Primer Name	Sequence	Use
10kb upstream Bik FW	5′-GTG TTG GGG CTG ATA GAC CA-3′	CHIP negative Control
10kb upstream Bik RV	5′-TCA GGG CAC TCT GGG AAA GA-3′	CHIP negative Control
Bik SNP-Chip-FW	5′-TAC AAA ACA CCA CTG GGC CT-3′	IRF1-Chip
Bik SNP-Chip-Rv (bikcons-r-nhe1	5′-ATA TAG CTA GCG ACA GGG ACC-3′	IRF1-Chip
(IRF1-EMSA-FW)IRF-780up-Optbiotin	5′-/Biosg/CTA GGG GGA AAC TGA AAC CCT-3′	IRF1-EMSA FW
IRF1-EMSA-Reverse	5′-AGG GTT TCA GTT TCG CCC TAG-3′	IRF1-EMSA-RV
P750A-FEMSA-FW (IRF-785Up-Gbiotin	5′-/Biosg/CTA GGG CCA GAC TGA AAA GCT-3′	P750G-EMSA-FW
Bik-G-EMSA-Reverse	5′-AGC TTT TCA GTC TGG CCC TAG-3′	P750G-EMSA-RV
P750g-FEMSA-FW (IRF-785Up-Abiotin	5′-/Biosg/CTA GGG CCA AAC TGA AAA GCT-3′	P750A-EMSA-FW
Bik-A-EMSA-Reverse	5′-AGC TTT TCA GTT TGG CCC TAG-3′	P750A-EMSA-RV
IRF1-EMSA-Compt-FW	5′-CAT GGG CGA AAC TAG AAC CCT-3′	
IRF1-EMSA-Compt-RV	5′-AGG GTT TCA GTT TCG CCC TAG-3′	
Bik-G-EMSA-Compet. FW	5′-CTA GGG CCA GAC TGA AAA GCT-3′	
Bik-G-EMSA-Compet. RV	5′-AGC TTT TCA GTC TGG CCC TAG-3′	
Bik-a-EMSA-Compet. FW	5′-CTA GGG CCA AAC TGA AAA GCT-3′	
Bik-A-EMSA-Compet. RV	5′-AGC TTT TCA GTT TGG CCC TAG-3′	

Figure S1

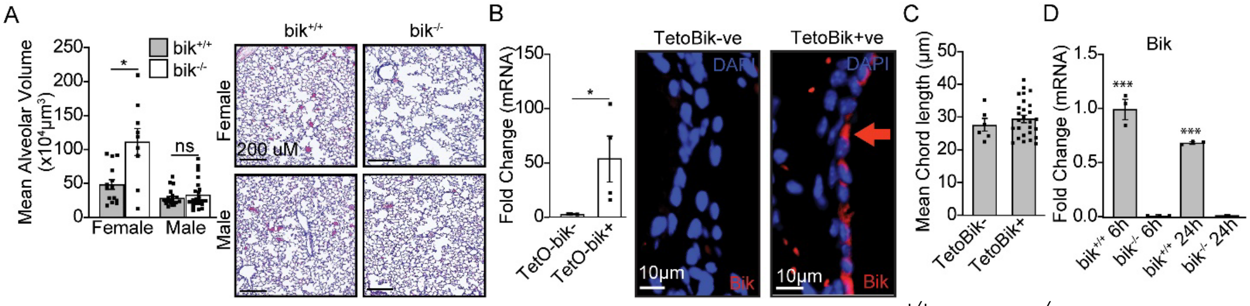

(A) Baseline weighted mean alveolar volume in female and male *bik*$^{+/+}$ and *bik*$^{-/-}$ mice at 56-80 weeks of age; n=10-14, N=2. **(B)** Airway-specific expression level of Bik mRNA and protein were analyzed by qRT-PCR and immunostaining, respectively. **(C)** Male TetoBik- and TetoBik- mice were kept with doxycycline containing water until the reach the age of 25 weeks and lung tissues were analyzed for changes in mean alveolar chord length. n=6 mice/group TetoBik- and 27 TetoBik+. **(D)** MAECs from *bik*$^{+/+}$ or *bik*$^{-/-}$ were plated in the presence of ROCK inhibitor medium and serum. ROCK inhibitor medium and serum were removed, and mRNA was analyzed 6 or 24 h later by qRT-PCR. n=3/group. N=number of repeats; n=sample size in a single experiment. , experimental replicates N=2. n=sample size in a single experiment; N=number of esperimental repeats. Two-tailed student t-test was used to compare between 2 groups and grouped results were analyzed using two-way analysis of variance. Data reported as mean ± SE; * $p < 0.05$, **$p < 0.01$, ***$p < 0.001$.

Figure S2

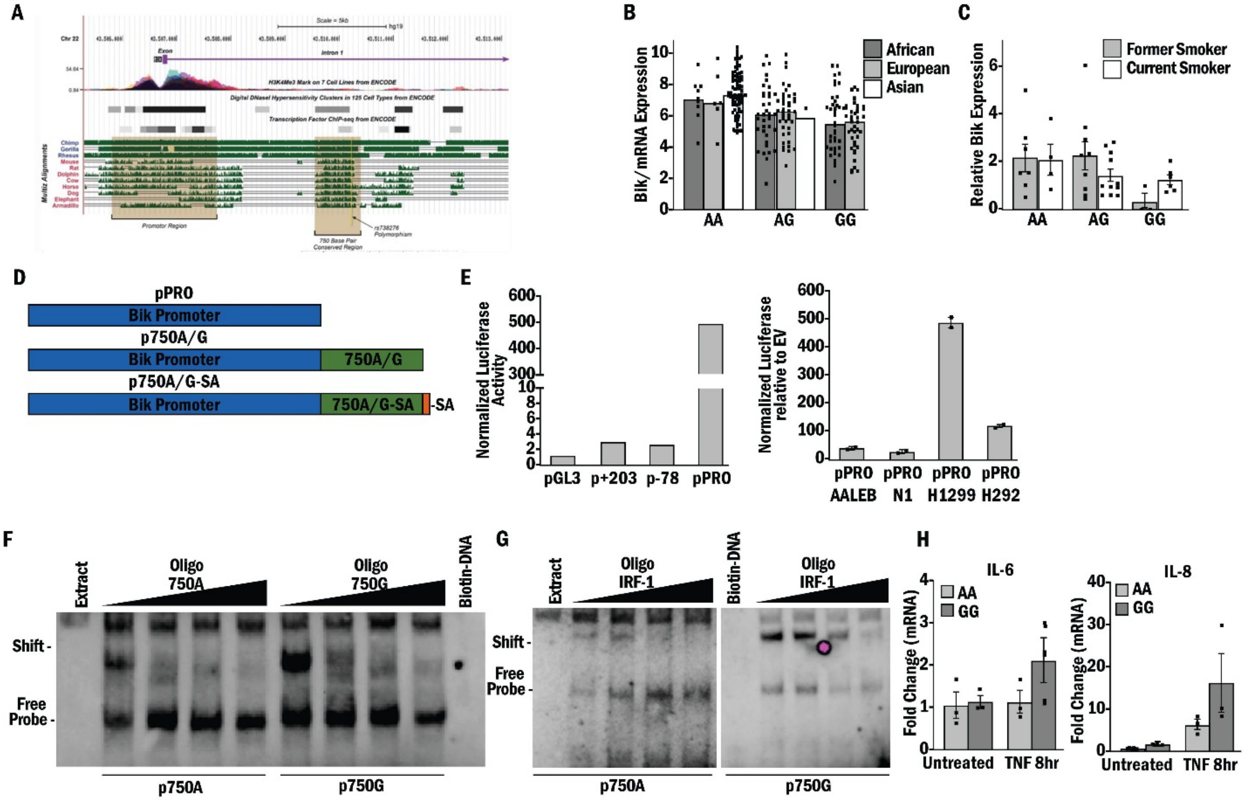

Legend to Supplement Figure 2: Bik expression in HAECs stratified by donor smoking status and ancestry. **(A)** The 750 bp intronic region of Bik gene is conserved from mouse to human. Figure was created using the UCSC Genome Browser (http://genome.ucsc.edu/index.html). Multi-alignment of select placental mammals is shown. Conservation values are displayed as a histogram (green) where the height is reflective of the score, and this score is based on alignment of 46 vertebrates. The location of rs738276 is indicated with a yellow vertical line. **(B)** HapMap microarray expression data for the 210 lymphoblastoid populations (YRI, CEU, JPT, CHB) was downloaded from https://www.ncbi.nlm.nih.gov/geo/geo2r/?acc=GSE6536. Expression data were analyzed individually for each major continental population. CHB and JPT groups represent the Asian population, while YRI and CEU represent African and European, respectively. Bars represent means ± SEM relative mean mRNA expression obtained from normalized microarray values. Mean group values were unlogged and further normalized to the GG genotype so that fold-differences. The number of samples assessed for each genotype is indicated on the x-axis. **(C)** HBECs from current (n=19) or former smokers (n=21) were obtained by bronchoscopy, cultured, and after 1 or 2 passage Bik mRNA was quantified by qPCR. Bars represent means ± SEM Ct values for Bik mRNA were normalized to the housekeeping CDKN1B mRNA, then group values were normalized to former smokers with GG genotype. Number of samples assessed for each genotype is indicated on the x-axis. **(D)** Comparison of promoter vectors with varying intron 1 proportions. The pGL3 basic vector, pPRO (-2000 to +980), p-78, and p+203 constructs were transfected (TransIT-2020) into H1299 human epithelial lung cancer cell line. To normalize the transfection efficiency across samples, the *renilla* firefly (RF) expression vector pRLTK was

included as an internal control. Cell lysates harvested at 24 h and RF and FF luciferase activity assessed using a dual-luciferase reporter assay system. The RF normalized pPRO, p-78 and p+203 values were further normalized to those of the pGL3 basic vector to indicate the strength of promoter activity. Bars denote mean±SEM (n = 3 independent experiments). The SNP rs738276 is located within a 750bp conserved region of intron 1 of the *BIK* gene. **(E)** The pPRO construct was transfected into AALEB, N1, H1299, and H292 cells and analyzed for luciferase activity. Significant luciferase activity was detected only in all four cell lines. **(F, G)** Promoter-luciferase constructs in the pGL3 basic vector were constructed with the *BIK* promoter (pPRO) and the 750 bp region encompassing rs738276 with the two variants downstream of the *BIK* promoter (p750A/G), and the p750A/G containing a splice acceptor (p750A/G-SA). Nuclear extracts from H1299 cells were prepared, and 10 µg of each was subjected to EMSA by using biotin-labeled p750A or p750G oligonucleotide probes or the IRF-1 oligo. Competition assays with **(F)** p750A or p750G and **(G)** IRF-1 oligo DNA were performed by adding a 0, 10-, 100-, and 1000-fold molar excess of unlabeled specific oligonucleotide probes to the reaction mixture. **(H)** NHBEs from people with AA or GG allele of Bik SNP were plated on 6-well plates. Cells were treated with 10 ng/ml TNF-alpha for 8 h and the mRNA expression levels of IL-6 and IL-8 were analyzed using qRT-PCR. Two-tailed student t-test was used to compare between 2 groups and grouped results were analyzed using two-way analysis of variance.

Figure S3

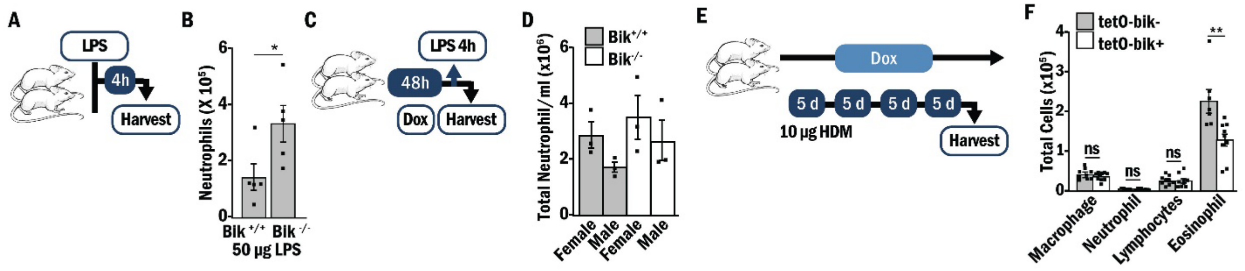

(A) $bik^{+/+}$ and $bik^{-/-}$ mice were instilled with 5 or 50µg LPS intranasally and neutrophil numbers were compared in the BAL fluid four hours later. **(B)** $bik^{+/+}$ and $bik^{-/-}$ mice were instilled with 50 µg LPS and BAL fluid was analyzed for neutrophil numbers 4 h later. n=5 mice/group.**(C)** Schematic for TetoBik- and TetoBik+ mice were placed on Dox diet for 48 h and subsequently instilled with 50 µg LPS and harvested 4 h later. (D) $bik^{+/+}$ and $bik^{-/-}$ mice were anesthetized with isoflurane and intranasally instilled with 5 µg LPS. Mice were euthanized 24 h later and BAL neutrophil numbers were quantified. n=3 male and 3 female/group. **(E)** Mice were sensitized to HDM on days 1 and 8 via intranasal instillation of 10 µg HDM and subsequently challenged with 10 µg HDM daily 5 days per week for 4 subsequent weeks. Mice were kept on Dox diet during the 4 weeks challenge. **(F)** Inflammatory cell numbers in the lavage fluid of mice sensitized with HDM on days 1 and 8 and challenged with HDM by IN instillation for 5 d/wk for 4 wks and kept on Dox diet during this time. n=9/group, experimental replicates N=2. Two-tailed student t-test was used to compare between 2 groups and grouped results were analyzed using two-way analysis of variance. Data reported as mean ± SE; *$p < 0.05$, ** $p < 0.01$, *** $p < 0.001$.

Figure S4

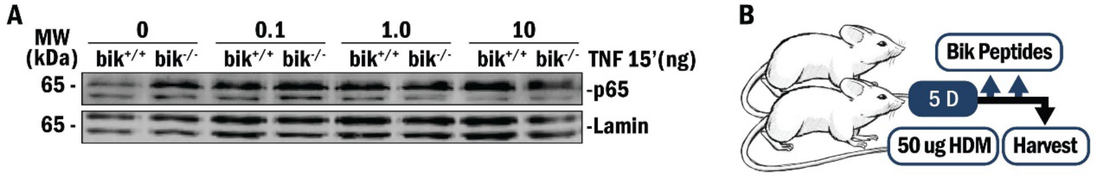

(A) Mouse Airway Epithelial Cells were isolated from female *bik*$^{+/+}$ and *bik*$^{-/-}$ mice and treated with 10 ng/ml TNF-α for 15 minutes. Nuclear lysates were analyzed by Western blot for the level of p65 protein. **(B)** *bik*$^{-/-}$ mice were instilled with 50 μg HDM intranasally daily for 5 consecutive days. On days 6 and 7, mice were intranasally treated with 10 μM of control TAT peptide, BH3 WT Bik peptide, or BH3 mutant Bik peptide. BAL fluids were analyzed for inflammatory cell numbers.

Figure S5

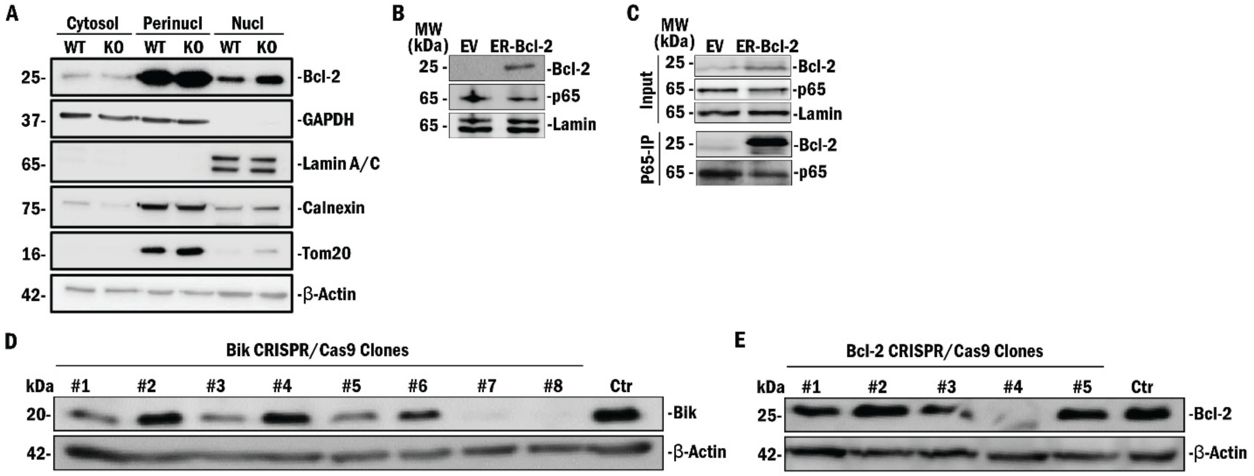

(A) Cytosolic, perinuclear and nuclear fractions were isolated from *bik*$^{+/+}$ and *bik*$^{-/-}$ MAECs. Protein lysates were analyzed for the expression levels of the indicated proteins by Western blot. GAPDH, Tom20, Calnexin, and Lamin A/C are shown as indicators for enrichment of the cytosolic, mitochondrial, ER, and nuclear fractions, respectively. **(B)** HEK293T cells were transfected with empty vector or ER-targeting Bcl-2 plasmids. After removing the cytosolic and perinuclear fractions, the nuclear fraction was subjected to Western blot. **(C)** HEK293T cells were transfected with empty vector or Bcl-2 expressing plasmids. The cytosolic fractions were immunoprecipitated with anti-p65 antibody and subjected to Western blot analysis. **(D)** HEK293T cells were infected with lentiviral vector for **(D)** BIK or **(E)** Bcl-2 CRISPR guide RNA and Cas9. BIK and Bcl-2 knockout clones were identified through selection using puromycin and expression level of BIK was compared between clones by Western blot**.** For the subsequent studies, clone #7 as Bik KO and clone #4 as Bcl-2 KO cells were used**.**

Figure S6

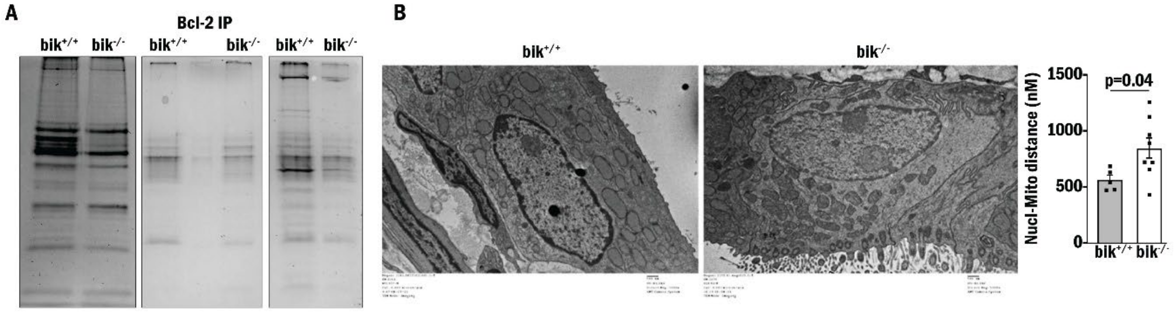

(A) Mouse airway epithelial cells from *bik*$^{+/+}$ and *bik*$^{-/-}$ mice were grown till 100% confluency. Nuclear fractions were isolated after removing the cytosolic and perinuclear fractions and run on SDS-PAGE gels. Protein bands were cut and submitted for proteomic analysis. **(B)** Localization of mitochondria and other organelles around the nuclei in the airway cells of *bik*$^{+/+}$ and *bik*$^{-/-}$ mice. Distances between the nuclei and adjacent mitochondria were measured using ImageJ software. N=5-8 per group. Two-tailed student t-test was used to compare between 2 groups. Data reported as mean ± SE.
